# Erythromycin reduces nasal inflammation by inhibiting immunoglobulin production, attenuating mucus secretion, and modulating cytokine expression

**DOI:** 10.1038/s41598-021-01192-8

**Published:** 2021-11-05

**Authors:** Ting-Ting Yen, Rong-San Jiang, Ching-Yun Chang, Chih-Ying Wu, Kai-Li Liang

**Affiliations:** 1 Department of Otolaryngology, Taichung Veterans General Hospital, 1650, Sec. 4, Taiwan Boulevard, Taichung, 40705 Taiwan; 2 Department of Medical Research, Taichung Veterans General Hospital, Taichung, Taiwan; 3 Department of Pathology and Medical Laboratory, Taichung Veterans General Hospital, Taichung, Taiwan; 4 Faculty of Medicine, National Yang-Ming Chiao-Tung University, Taipei, Taiwan; 5 School of Medicine, Chung Shan Medical University, Taichung, Taiwan

**Keywords:** Diseases, Medical research, Pathogenesis

## Abstract

Allergic rhinitis (AR) and chronic rhinosinusitis (CRS) share some similar pathological mechanisms. In current study, we intend to investigate the impact of AR on CRS. In addition, we explored the efficacy of erythromycin (EM) treatment on CRS mice with or without AR (CRSwoAR, CRSwAR). Study subjects were divided into control, CRSwoAR, and CRSwAR groups. Experimental mice were divided similarly into control, CRSwoAR, and CRSwAR groups. In addition, CRS mice were treated with EM at 0.75, 7.5, or 75 mg/kg or with dexamethasone (Dex) at 1 mg/kg. In our results, allergy exacerbates inflammation that was evident in nasal histology and cytokine expression both in patients and in mice with CRS. Dex 1 mg/kg, EM 7.5 or 75 mg/kg treatments significantly inhibited serum IgE and IgG2a in CRS mice. EM-treated CRS mice had significantly elevated IL-10 levels and had a reversal of Th-1/Th-2 cytokine expression in nasal-associated lymphoid tissue. MUC5AC expressions were significantly reduced in the 7.5 or 75 mg/kg EM-treated mice compared with untreated mice. EM showed inhibitions on immunoglobulin production and mucus secretion stronger than Dex. We concluded that comorbid AR enhanced inflammation of CRS. EM and Dex treatments showed similar anti-inflammatory effects on CRS but through partly different mechanisms.

## Introduction

Allergic rhinitis (AR) and chronic rhinosinusitis (CRS) are the two most prevalent upper airway diseases. AR is an immunoglobulin E (IgE)-mediated and T helper (Th-2) immune response related nasal inflammation. CRS is defined as inflammation of sinonasal mucosa which lasts for more than 12 weeks^1^. The pathophysiology of CRS is complex including anatomic variation, microbe infection, biofilm, allergy, immunodeficiencies, disturbance of epithelial barrier function, impaired ciliary function, or genetic factors^2^. Allergic rhinitis and rhinosinusitis are frequently associated and share some similar pathological mechanisms^3,4^. However, there are no controlled studies about the impact of allergy on pathophysiology of CRS, nor well-designed studies associated the treatment of allergy affect CRS outcome^5^. Wilson et al. conducted an evidence-based review and concluded that the association between allergy and CRS remained unclear^6^.

Airway mucosa is the first line of defense protecting the human body from environmental pathogens, allergens, and irritants. Regarding functional airway barriers, the mucociliary escalator, epithelial integrity, and secretary antimicrobial peptides are their three primary components^7^. Mucociliary clearance helps to trap invading foreign particles before removing them from the airway. Epithelial barrier is formed by epithelium, intercellular tight and adherens junctions. Together they maintain barrier integrity by controlling the paracellular permeability. Mucus hypersecretion is a major consequence of chronic airway diseases. Previous clinical studies found MUC5AC expression is important in CRS pathophysiology^8,9^. Disruption of airway epithelial junctions is also a pathophysiologic finding of AR^10,11^. Defects in the sinonasal epithelial barrier, increased exposures to pathogens, dysregulation of the host immune system and mucociliary clearance all of which are considered important in the pathophysiology of chronic rhinosinusitis^12,13^.

Underlying inflammatory mechanisms are no doubt the main etiology of CRS^14,15^. Recently, researchers recommended new classification of CRS patients based on inflammatory patterns (endotype) rather than the clinical presentation (phenotypes)^16,17^. Bachert et al. proposed the CRS classification according to Th-cell populations and Th-related cytokines^17,18^. Update guidelines for rhinosinusitis recommend utilizing the Th-1 (IFN-γ, TNF-α), Th-2 (IL-4, -5, 13, ECP), and Th-3 (IL-17A) biomarkers to defining subtypes of CRS^1,2^. Patients of CRS comorbid with allergy display immune responses comparable to those underlied by Th-2 inflammation.

Nasal steroid and long-term macrolide are current standard treatment for CRS^1^. Steroid is well-known for its broad-spectrum anti-inflammatory effects but the effects of macrolides on CRS remained uncertain. Macrolides treatment experiences come from the dramatic lifesaving outcomes from panbronchiolitis^19^, then extend their use to many chronic airway diseases. Despite the wide use of macrolides in treating CRS, updated rhinosinusitis guidelines recommended more studies are needed to clarify their effects^1,2^. The efficacy of macrolides might come from both antibiotic and non-antibiotic effects. Mechanisms proposed on the non-antibiotic effects of macrolides on CRS include the inhibition of biofilm formation, enhancement of mucociliary clearance, modulation of cytokine production, and promotion of neutrophil apoptosis^20,21^. Systematic reviews and meta-analyses reported macrolides are beneficial only on some but not all CRS patients^5,22^. Seresirikachorn et al*.*^22^ concluded that macrolides are beneficial in treating patients with CRS without nasal polyps as opposed to CRS with nasal polyps. In the study by Perić et al.^23^ nasal polyposis patients with or without AR received clarithromycin for 8 weeks. They found that immunomodulatory effects on cytokines were different between the two groups. The purpose of this study was to investigate the impact of AR on pathophysiology of CRS. In addition, we determined the efficacy of the erythromycin (EM), a 14-membered macrolide, on CRS mouse models with or without AR.

## Methods

### Rhinosinusitis subjects

Sinonasal tissues of CRS were obtained from patients after endoscopic sinus surgery. Control tissues were from patients without rhinosinusitis but had undergone septum or turbinate surgery. The CRS diagnosis was based on typical symptoms over 12 weeks and the results of endoscopy and computed tomography (CT), in line with the criteria of European Position Paper on Rhinosinusitis and Nasal Polyps 2020^1^. Enrolled subjects underwent sinus surgery due to medical treatment failure. All CRS patients received CT before surgery. CT images were graded according to the Lund and Mackay staging system with a total score range from 0 to 24^24^. Patients were diagnosed with AR according to specific IgE tests and clinical history for at least 2 years on having allergic symptoms when contacted with allergens and irritants. Enrolled subjects were divided into three groups: control, CRS without allergic rhinitis (CRSwoAR), and CRS with allergic rhinitis (CRSwAR) groups. In this study, we did not recruit asthmatic patients.

### Histologic characteristics of human sinonasal tissues

Tissues were fixed with buffered formalin, embedded in paraffin, and cut into slices. Hematoxylin and eosin (H&E) and immunochemistry (IHC) stainings were performed to evaluate histological features, changes in protein expression at tight and adherens junctions, and mucus production. The primary antibodies for evaluating the epithelium inter-cellular junction were claudin-1(Catalog No. 51–9000, polyclonal antibody, Thermo Fisher, IL, USA) and e-cadherin (catalog No. GTX100443, GeneTex, Irvine, CA, USA). Periodic acid-Schiff (PAS) staining (ScyTek Laboratories, Inc., Logan, UT, USA) was used to evaluate mucus production.

### Inflammatory cytokines/chemokines expression of human sinonasal tissues

Nasal mucosal tissues (100 mg/sample) were grinded and homogenized with protein extraction buffer (Thermo Scientific, Rockford, IL, USA). Cytokine expressions of IL-4, IL-5, IL-6, IL-10, IL-17A, and IFN-γ in the nasal tissue homogenates (50 µl, protein concentration of 5 mg/ml) were determined using Bio-Plex®.

### Western blotting of human sinonasal tissues

Equal amounts of nasal mucosal proteins (40 μg) extracted by the aforementioned method were subjected to electrically separated in 10% polyacrylamide gel, and then transferred to polyvinylidene difluoride membrane. Because of limited amount of nasal tissue retrieved from the experimental mice, we cut the polyvinylidene difluoride membrane, before blocking and incubating with individual primary antibody. The membrane was incubated with the following primary antibodies: claudin-1 (1:1000, catalog No. E-AB-30939, Elabscience, Houston, USA), e-cadherin (1:2000, catalog No. GTX100443, GeneTex, Irvine, CA, USA), MAC5AC (1:100, clone No. 2-11M1, abcam, Cambridge, UK), and GAPDH (1:5000, catalog No. 60004-1-1 g, Proteintech, Rosemont, USA). On the following day, the membranes were incubated with HRP-conjugated secondary antibody, followed by electrochemiluminescent detection (Millipore Billerica, MA, USA). The density of each protein band was scanned using ImageJ Software, version 1.46r (National Institutes of Health, Bethesda, MD) and compared in densitometry.

### Allergic rhinitis and chronic rhinosinusitis mouse models

Female BALB/c mice at 6–8 weeks of age were divided into 3 groups: control, CRSwoAR, and CRSwAR. On days 0, 7, and 14, AR mice were sensitized each with an intraperitoneal injection of 4 μg house dust mite (HDM, Indoor Biotechnologies Ltd, Cardiff, UK) mixed with 40 μg aluminum hydroxide gel adjuvant (Invitrogen, San Diego, CA). From days 22 to 26, animals received intranasal challenges with 4 μg HDM. Non-AR mice received sham sensitization. Mice underwent nasal surgery to induce chronic rhinosinusitis. In brief, under anesthesia, a 1-cm incision was made over the mouse head, and a 3 mm hole was drilled on the skull to reach nasal cavity on one side. Gelfoam pledgets (Johnson & Johnson, Gargrave, Skipton, UK) were inserted into the nasal cavity to induce local inflammation. The scalp wounds were approximated with sutures at the end of the procedure. With the protocols, experimental AR and CRS could be reliably induced with evidence of histology and biomarkers in our previous study^25^. Mice were sacrificed on day 27. Mouse rhinosinusitis lasting > 4 weeks was regarded, in the literature, as a chronic disease model^26^.

Blood samples, nasal mucosa, and nasal-associated lymphoid tissue (NALT) were collected for analyses. Sera were used to measure levels of IgE and IgG using commercial mouse IgE and IgG ELISA kits according to manufacturer’s instructions (BD Pharmingen, San Diego, CA, USA). Mice nasal tissue sections were examined after H&E and IHC stainings including the following: PAS (ScyTek Laboratories, Inc., Logan, UT, USA), claudin-1 (51-9000, Thermo Fisher Scientific Inc., IL, USA) and e-cadherin (catalog No. GTX100443, GeneTex, Irvine, CA, USA). Tissue eosinophilia was determined by counting eosinophil numbers of 5 high power fields (magnification × 400)^27^. Nasal mucosa and NALT were then grinded and homogenized with the protein extraction buffer (Thermo Scientific, Waltham, MA, USA). Protein extracts from nasal mucosa were used to determine protein expressions of claudin-1, e-cadherin, MUC5AC, and GAPDH (primary antibodies supplied by Elabscience, Houston, USA; BD Pharmingen, San Diego, CA, USA; GeneTex, Irvine, CA, USA; abcam, Cambridge, UK, and Proteintech, Rosemont, USA). Proteins extracted from NALT (50 μg, concentration 1 mg/ml) were used to determine with the Bio-Plex® (Bio-Rad, Hercules, CA, USA) local levels of cytokines (IL-4, IL-5, IL-6, IL-10, IL-17A, and IFN-γ).

### Erythromycin and dexamethasone treatments on CRS mice

CRSsAR mice (N = 43) and CRSwAR mice (N = 58) received days 23 to 25 intraperitoneal injections of either dexamethasone 1 mg/kg (Dex) or erythromycin (EM, 0.75, 7.5, or 75 mg/kg).

### Statistical analyses

Chi-Square test was used to compare the categorical data. Mann–Whitney U test was used to compare continuous data between two groups. Kruskal–Wallis test was used to compare 3 or more groups. Then Dunn’s multiple comparisons test was used for post hoc examination of between-group differences. Statistically significance was set at P < 0.05. Statistical analyses were performed using GraphPad Prism 9.2.0 for Mac (GraphPad Software, San Diego, CA, USA) and IBM SPSS 20.0 (IBM Corp. Armonk, NY, USA).

### Ethics

The Institutional Review Board of Taichung Veterans General Hospital approved the present study on human subjects (CE17143A). The research was performed in accordance with relevant human study guidelines and regulations. Inform consent was obtained from each study subject.

The animal use protocol and experimental procedures have been reviewed and approved by the Institutional Animal Care and Use Committee of Taichung Veterans General Hospital (La-1071579). All experiments were performed following the recommendations of guidelines. Current study is reported in accordance with ARRIVE guidelines^28^.

## Results

### Human study

**Figure 1:**
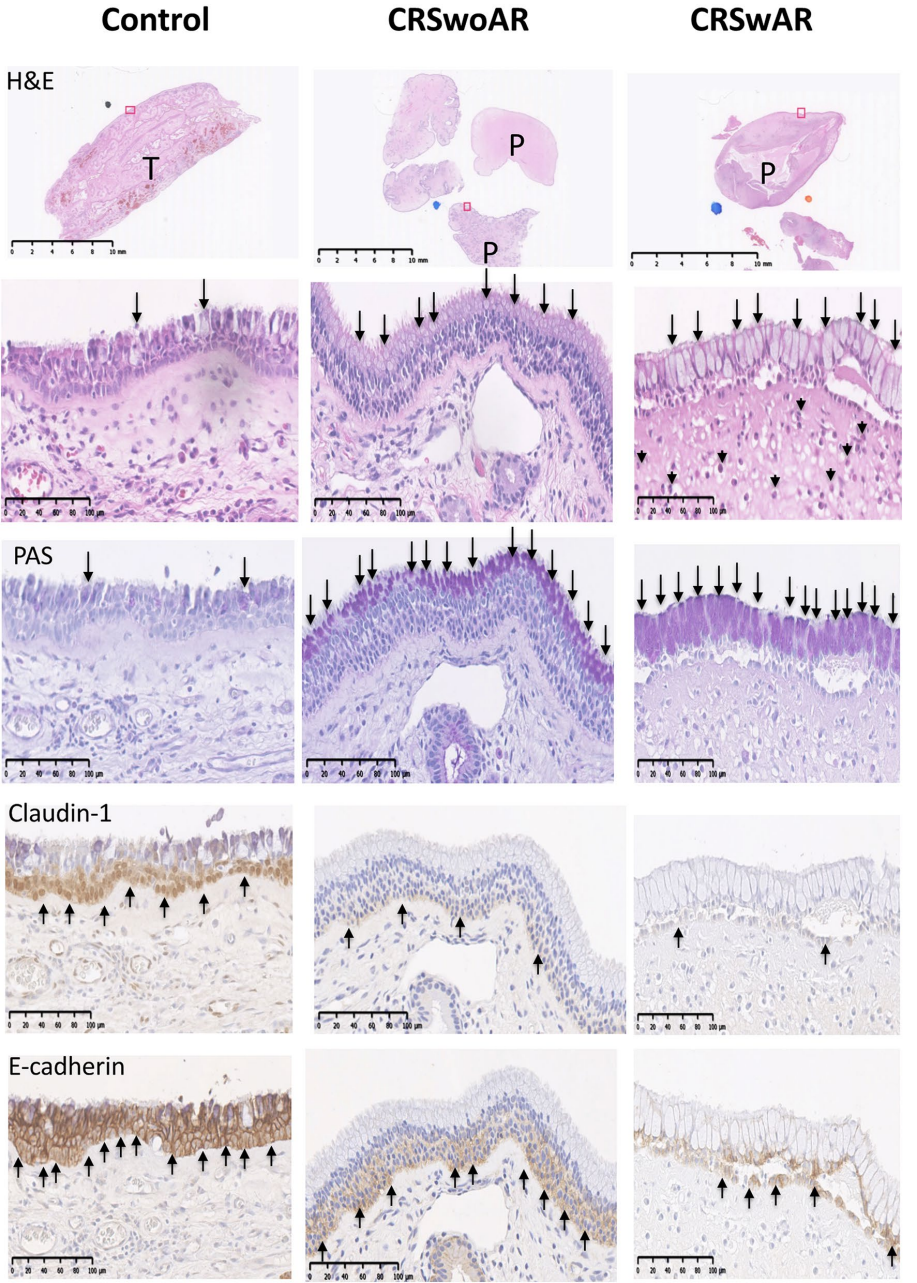
Representative images showing histological features of nasal tissues of various groups. Increased number of goblet cells, increased mucus production, decreased claudin-1 and e-cadherin staining are found in tissue sections of CRSwoAR and CRSwAR. Control: without rhinosinusitis; CRSwoAR: chronic rhinosinusitis without allergic rhinitis; CRSwAR: chronic rhinosinusitis with allergic rhinitis; T: inferior turbinate; P: nasal polyp; H&E: hematoxylin and eosin staining; PAS: periodic acid-Schiff staining. Arrow heads mark the eosinophils; arrows mark the representative goblet cells, PAS staining, e-cadherin and claudin-1 staining cells.

Their clinical characteristics of CRS patients are listed in Table 1. Sinonasal tissues from the CRSwAR group showed higher mucosal eosinophilia and globlet cell hyperplasia when compared with the other two groups. In addition, in nasal epithelia of the CRSwAR group immunochemistry stainings to of claudin-1 and e-cadherin were less compared with the other two groups. Representative images of H&E and IHC stainings are shown in Fig. 1.

**Table 1:** Clinical characteristics of study subjects.

Group	Control	CRSwoAR	CRSwAR	P
Case number	16	26	21	
Male/female	13/3	21/5	7/14	0.001* ^a^
Age (range), years	38.5 (20–72)	40 (20–65)	43 (20–80)	0.729^b^
Smoker	4	5	4	0.883^a^
Nasal polyp	N/A	21	16	0.737^c^
CT score (range)	N/A	14 (4–24)	11.5 (2–22)	0.233^d^

CRSwoAR, chronic rhinosinusitis without allergic rhinitis; CRSwAR, chronic rhinosinusitis with allergic rhinitis; CT, computed tomography.

^a^Chi-square test.

^b^Kruskal–Wallis test.

^c^Fisher’s exact test.

^d^Mann–Whitney U test.

*P < 0.05.

**Figure 2:**
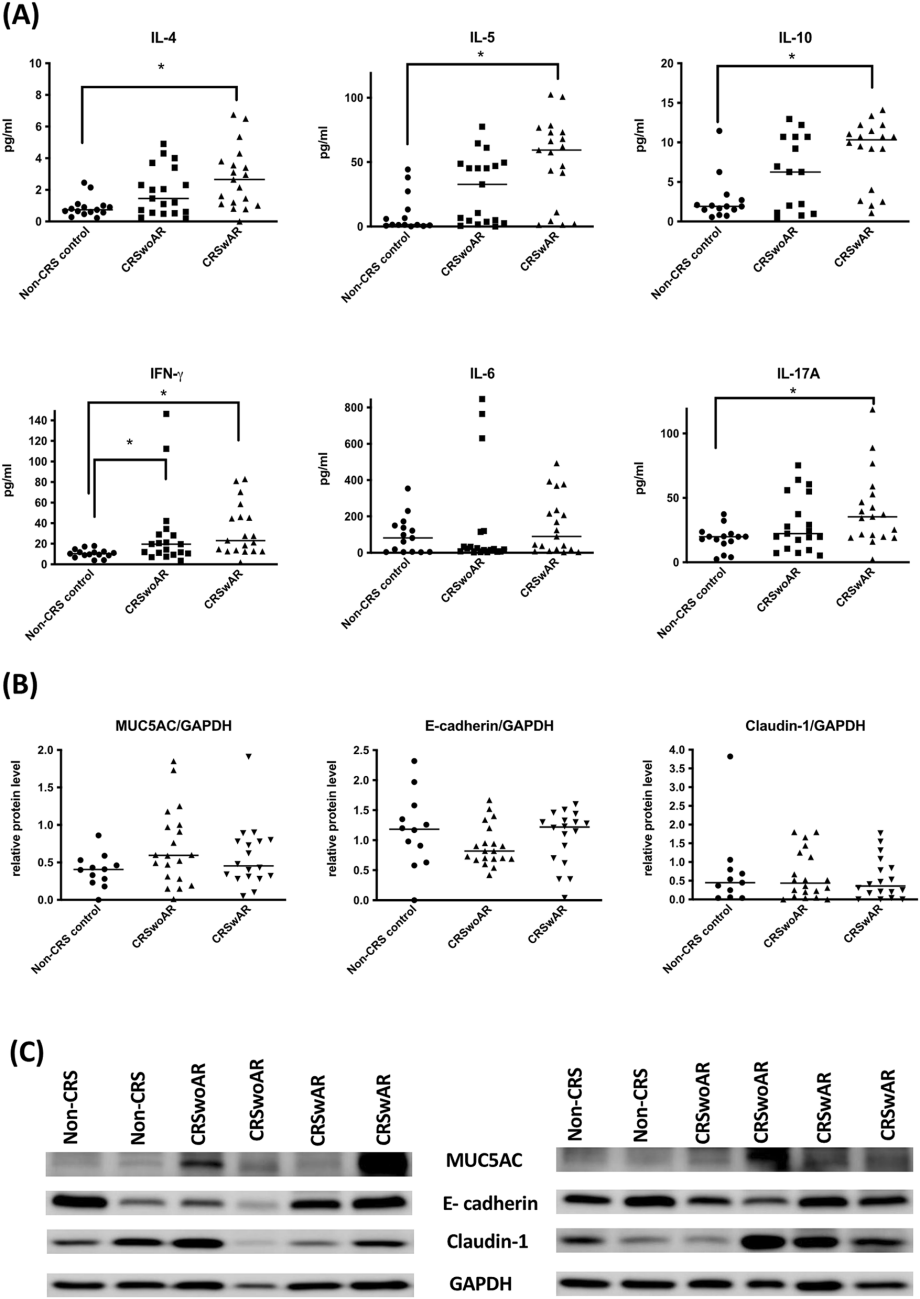
(**A**) Cytokine levels in tissue homogenates from nasal mucosa of study subjects, horizontal lines represent medium of each group. Control: without rhinosinusitis; CRSwoAR: chronic rhinosinusitis without allergic rhinitis; CRSwAR: chronic rhinosinusitis with allergic rhinitis. (**B**) Comparison of the levels of protein expression among the three groups. (**C**) Representative samples of protein expression of study subjects. Because of limited amount of nasal tissue, we cut the polyvinylidene difluoride membrane before blocking and incubating with individual antibody. Full length images of western blotting are shown in Supplementary Figs. S1 & S2. Horizontal line represents medium of each group. *P < 0.05.

Cytokines levels of IL-4, IL-5, IL-10, and IL-17A in tissue homogenates from patients with CRSwAR were significantly higher than those of controls (P = 0.0027, 0.0004, 0.0015, and 0.0205, respectively). In addition, IFN-γ levels were significantly higher in both CRSwoAR and CRSwAR than controls (CRSwoAR vs. control: P = 0.0474; CRSwAR vs. control, P = 0.0005) (Supplementary Table S1; Fig. 2A). Western blotting showed a protein expression tendency of increasing MUC5AC and decreasing e-cadherin and claudin-1 in nasal mucosa from CRS patients when compared with controls (Supplementary Table S2; Fig. 2B,C). Nevertheless, the apparent differences were not statistically significant between groups (MUC5AC, e-cadherin, claudin-1, P = 0.1899, 0.3014, and 0.8877, respectively).

### Mice models of rhinosinusitis with or without allergy

**Figure 3:**
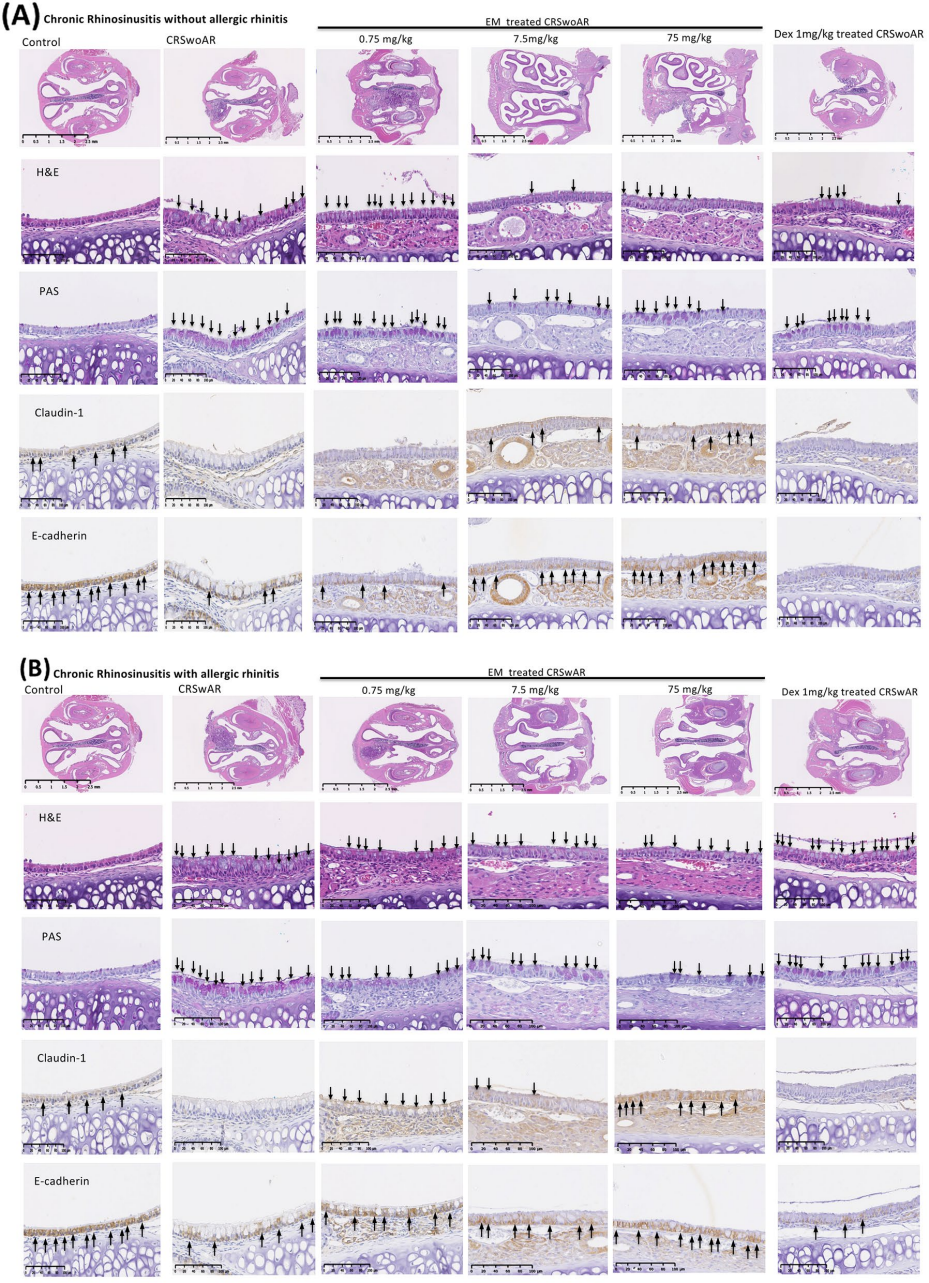
Representative images showing histological features of nasal tissues in (**A**) chronic rhinosinusitis mice without allergic rhinitis and (**B**) chronic rhinosinusitis mice with allergic rhinitis. Increased number of goblet cells, increased mucus production, decreased claudin-1 and e-cadherin staining are found in tissue sections of CRSwoAR and CRSwAR. Control: without rhinosinusitis; CRSwoAR: chronic rhinosinusitis without allergic rhinitis; CRSwAR: chronic rhinosinusitis with allergic rhinitis; EM: erythromycin; Dex: dexamethasone; H&E: hematoxylin and eosin staining; PAS: periodic acid-Schiff staining. Arrow heads mark the eosinophils; arrows mark the representative goblet cells, PAS staining, e-cadherin and claudin-1 staining cells.

**Figure 4:**
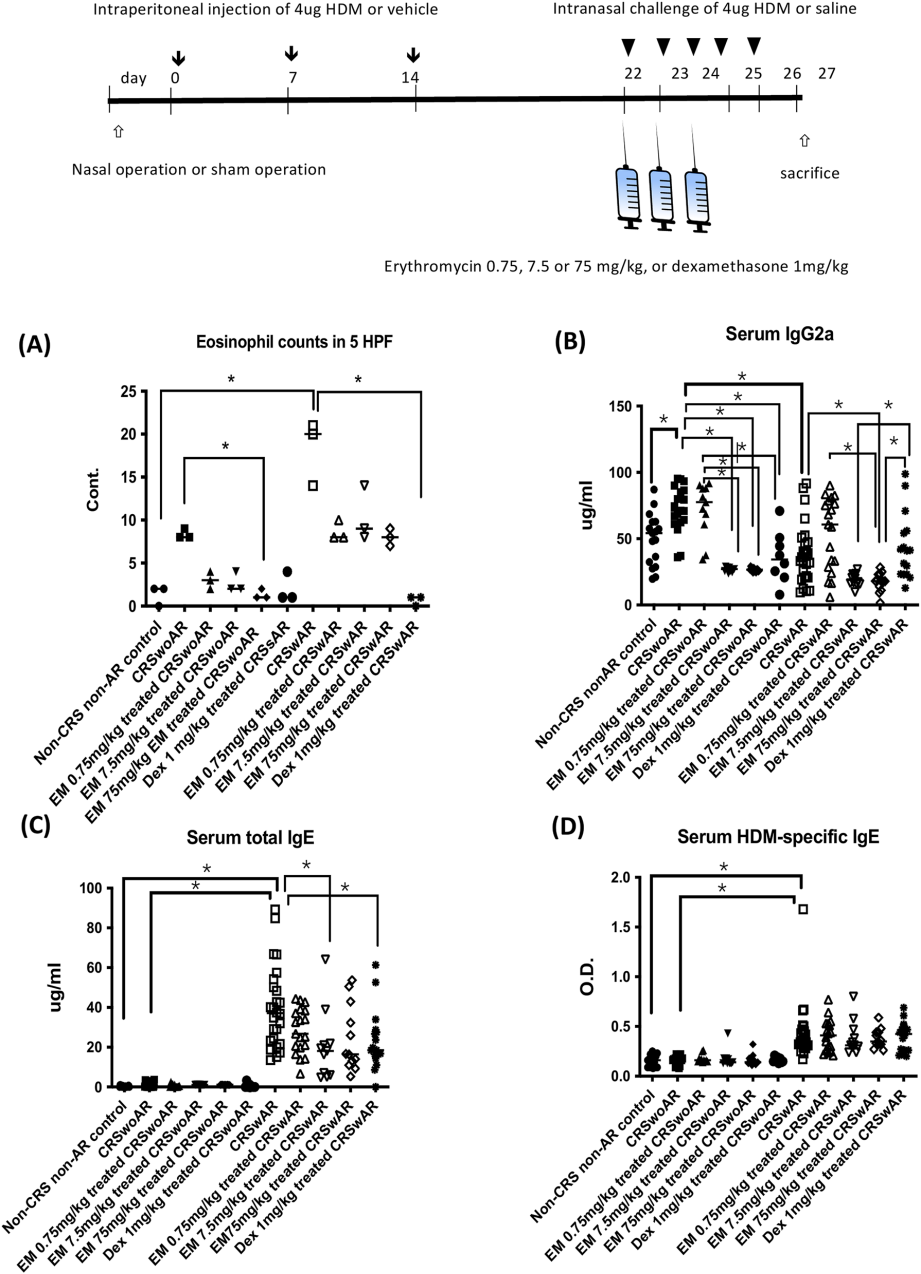
Protocol of mice experiments. (**A**) total eosinophil counts of 5 high power views in lamina propria of nasal mucosa. (**B**–**D**) IgG2a, IgE, and house dust mite specific IgE levels of experimental mice. CRSwoAR: chronic rhinosinusitis without allergic rhinitis; CRSwAR: chronic rhinosinusitis with allergic rhinitis; EM: erythromycin; Dex: dexamethasone. Horizontal line represents medium of each group. *P < 0.05.

In nasal epithelia of diseased mice, H&E staining showed greater mucosal cellular infiltration, globlet cell hyperplasia, lower epithelial IHC stainings for claudin-1 and e-cadherin, and higher PAS staining (Fig. 3A,B). There were significantly increased mucosal eosinophils found in CRSwAR mice (Supplementary Table S3; Fig. 4A, control vs. CRSwAR, P = 0.0205). AR mice showed significantly higher levels of total and HDM-specific IgE when compared with the non-AR mice (Supplementary Table S4, control vs. CRSwAR, CRSwoAR vs. CRSwAR, both P < 0.0001). CRSwoAR mice had significantly higher serum IgG2a levels compared with controls or CRSwAR mice (control vs. CRSwoAR, P = 0.0179, and CRSwoAR vs. CRSwAR, P < 0.0001) (Supplementary Table S4; Fig. 4B–D).

**Figure 5:**
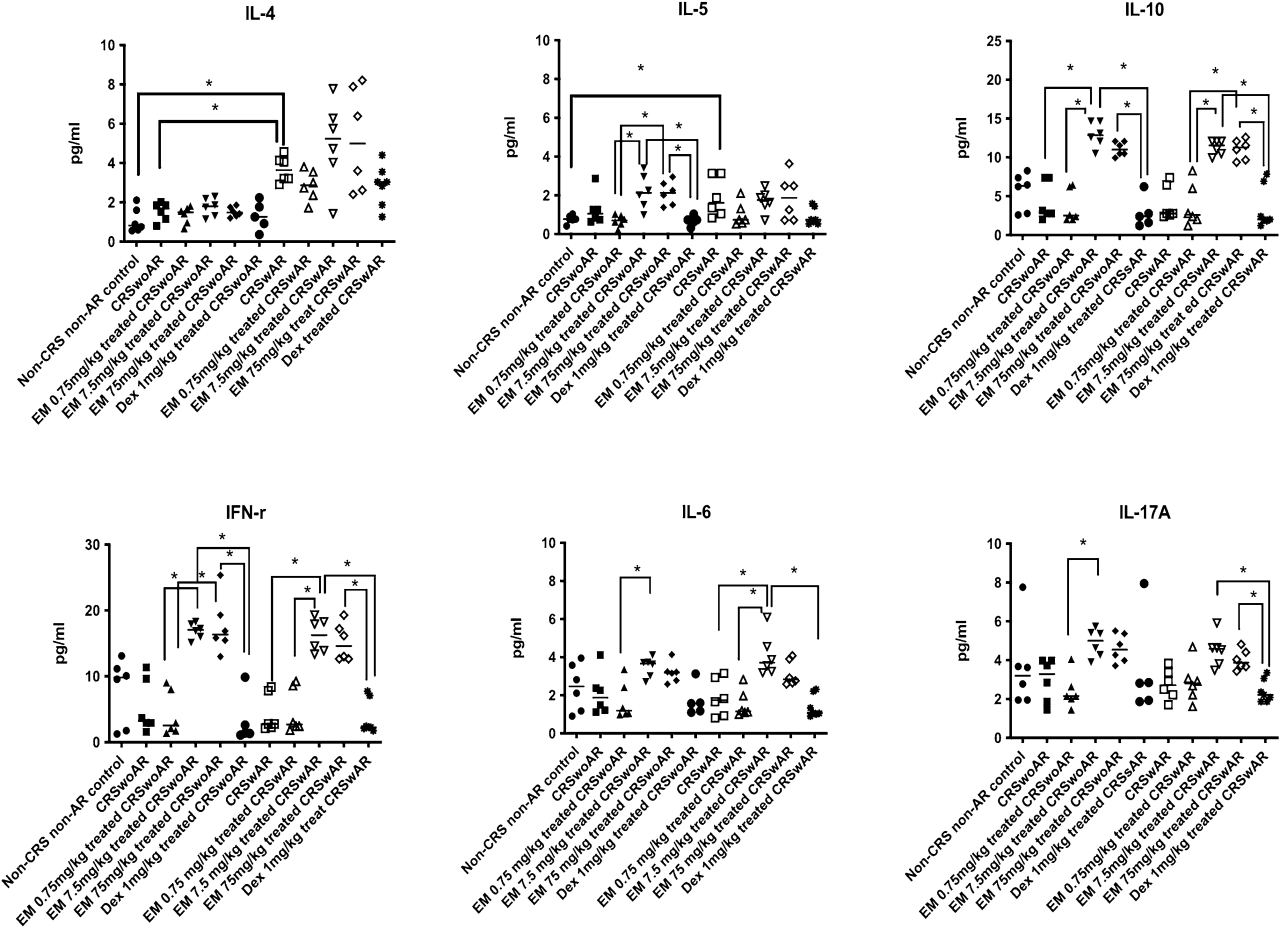
Cytokine levels in tissue homogenates taken from nasal-associated lymphoid tissues in the experimental mice. CRSwoAR: chronic rhinosinusitis without allergic rhinitis; CRSwAR: chronic rhinosinusitis with allergic rhinitis; EM: erythromycin; Dex: dexamethasone. Horizontal line represents medium of each group. *P < 0.05.

**Figure 6:**
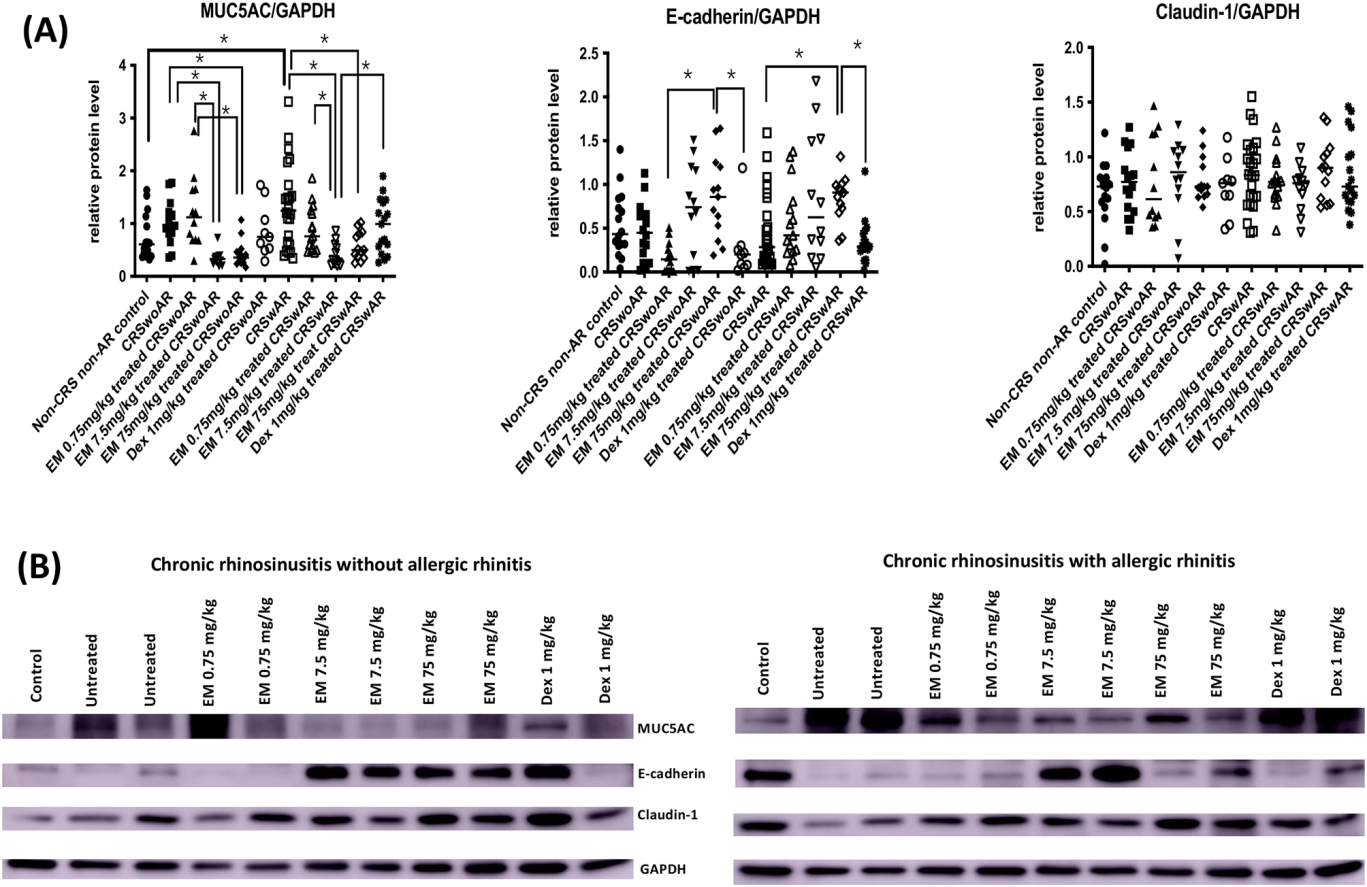
(**A**) Relative protein expressions of MUC5AC, e-cadherin, and claudin-1 in nasal mucosa of experimental mice (**B**) Representative results of western blotting. Because of limited amount of nasal tissue, we cut the polyvinylidene difluoride membrane before blocking and incubating with individual antibody. Full length images of western blotting are shown in Supplementary Figs. S3 & S4. CRSwoAR: chronic rhinosinusitis without allergic rhinitis; CRSwAR: chronic rhinosinusitis with allergic rhinitis; EM: erythromycin; Dex: dexamethasone. Horizontal line represents medium of each group. *P < 0.05.

Allergic mice had significantly higher levels of IL-4 and IL-5 levels in tissue homogenates from NALT compared with controls (IL4: CRSwAR vs. control, P = 0.0024, CRSwAR vs. CRSwoAR, P = 0.0384; IL-5: CRSwAR vs. control, P = 0.0279) (Supplementary Table S5; Fig. 5). MUC5AC protein expression appeared higher in nasal tissues of the CRSwoAR or CRSwAR mice than that of controls. The difference between CRSwAR and controls was statistically significant (CRSwAR vs. Control, P = 0.0144). There was a tendency of decreased e-cadherin protein expression in rhinosinusitis mice with or without AR but without significant between group differences (Supplementary Table S6; Fig. 6).

### Effect of erythromycin or dexamethasone treatment on experimental mice

After treatments with 7.5 or 75 mg/kg EM, or with Dex, we found reduced cellular infiltrations in nasal histology of CRS mice (with or without AR). Marked reduction in mucus production was especially noted in CRS mice receiving EM treatments. In addition, we found stronger e-cadherin stainings in nasal tissues of EM-treated mice when compared with untreated mice (Fig. 3A,B). CRSwoAR mice treated with 75 mg/kg EM had significantly reduced mucosal eosinophilia as compared to untreated CRSwoAR mice (P = 0.0439). Dex-treated CRSwAR mice had significantly reduced mucosal eosinophilia than untreated CRSwAR mice as well (P = 0.0105) (Supplementary Tables S3 & S4; Fig. 4A).

CRSwoAR mice had significantly lower serum levels of IgG2a after treatment with 7.5 or 75 mg/kg EM (both P < 0.0001) or after 1 mg/kg Dex (P = 0.0229) when compared with untreated mice (Supplementary Table S7). CRSwAR mice treated with 75 mg/kg EM also significantly lowered their IgG2a levels when compared with untreated mice (P = 0.0272). In addition, 7.5 and 75 mg/kg EM-treated CRSwAR mice showed significantly lower serum levels of IgG2a compared with Dex-treated CRSwAR mice (P = 0.0167 and = 0.0075, respectively) (Supplementary Table S8; Fig. 4B–D). CRSwAR mice treated with 7.5 mg/kg EM or 1 mg/kg Dex significantly lowered their serum levels of total IgE when compared with the untreated CRSwAR mice (Supplementary Table S8, P = 0.0141 and = 0.0433, respectively).

Cytokine expressions in NALT homogenates demonstrated an immunomodulation effect of EM (Supplementary Tables S9 & S10; Fig. 5). CRSwoAR mice treated with 7.5 mg/kg EM had significantly elevated IL-10 levels in NALT homogenates than untreated CRSwoAR mice (P = 0.0387). In addition, EM-treated mice had significantly higher IL-10 levels than Dex-treated mice (CRSwoAR: EM 7.5 mg/kg vs. Dex, P = 0.0021; EM 75 mg/kg vs. Dex, P = 0.0417; CRSwAR: EM 7.5 mg/kg vs. Dex, P = 0.0043; EM 75 mg/kg vs. Dex, P = 0.0070). Interestingly, a reversal of Th-1/Th-2 cytokine expressions was found in the EM-treated mice. NALT IL-5 levels increased in the 7.5 and 75 mg/kg EM-treated CRSwoAR mice, and both were significantly higher than the Dex-treated mice (P = 0.0395, and 0.0356, respectively). In the CRSwAR group, NALT IL-6 and IFN-γ levels significantly rose in the 7.5 mg/kg EM-treated than the untreated mice, as well as the Dex-treated mice (IL-6: EM 7.5 mg/kg vs. untreated, P = 0.0471, EM 7.5 mg/kg vs. Dex, P = 0.0028; IFN-γ: EM 7.5 mg/kg vs. untreated, P = 0.0281, EM 7.5 mg/kg vs. Dex, P = 0.0020).

The western blotting results are shown in Fig. 6 and Supplementary Tables S11 & S12. MUC5AC protein expressions were significantly reduced in the 7.5 or 75 mg/kg EM-treated CRSwoAR mice compared with untreated mice (P = 0.0003 and = 0.0032, respectively). Similarly, CRSwAR mice receiving 7.5 or 75 mg/kg EM treatment had significantly lower nasal MUC5AC protein expressions when compared with untreated mice (P < 0.0001 and = 0.0134, respectively). In addition, we found significant differences in MUC5AC protein expressions between 7.5 mg/kg EM-treated and Dex-treated CRSwAR mice (P = 0.0190). Both CRSwoAR and CRSwAR mice showed higher e-cadherin expressions in nasal tissues after treatments with 7.5 or 75 mg/kg EM. No significant difference was found between EM-treated and untreated groups, but EM-treated CRS mice had significantly higher e-cadherin expression than Dex-treated mice (CRSwoAR: EM 75 mg/kg vs. Dex, P = 0.0293; CRSwAR, EM 75 mg/kg vs. Dex, P = 0.0105). Regarding claudin-1, there were no significant differences among the experimental groups. In brief, both erythromycin and dexamethasone treatment reduced nasal inflammation in treated mice, but through partly different mechanisms.

## Discussion

Our CRS subjects, with or without AR, showed similar CT grading scores and incidence of polyposis. Nevertheless, CRSwAR patients showed in their nasal tissues, signs of stronger mucosal inflammation, higher mucus production, higher Th-2 cytokines expression, and poorer epithelial tight junction protein expression when compared with those without AR. Xing et al.^29^ conducted a study investigating the effect of AR on nasal mucosa remodeling with nasal tissues taken from CRSwoAR and CRSwAR patients. They concluded that AR could enhances tissue remodeling process in CRS. Our results are consistent with CRS endotype being a better predictor than phenotype on disease severity. Based on the underlying inflammatory patterns, the classification of CRS patients with endotype could choose precise therapeutic strategy better for patients, and hence a better prediction of treatment outcome^30^.

Both EM and Dex effectively reduced serum levels of IgE in allergic mice. Furthermore, treatments both reduced serum IgG2a production in either CRSwoAR or CRSwAR mice. Only two randomized controlled studies have so far been conducted relating macrolides treatment on CRS patients measuring IgE levels at the time of enrollment: Wallwork et al.^31^ reported that macrolides have benefits especially on CRS patients with low levels of IgE, but Hexel et al.^32^ did not found such advantage in their similar patients. Eosinophilic inflammation is a major hallmark of AR and CRS with nasal polyps. In CRSwoAR mice, EM treatment at a dose of 75 mg/kg demonstrated an inhibitory effect of tissue eosinophilia. Consistent with our present results, macrolides could have a role in treating IgE-medicated allergic airway diseases despite reported studies favored benefits on low-IgE CRS subjects.

We had conducted a study comparing the effects of intranasal steroid or EM towards rhinosinusitis patients^33^. Results of aforementioned study demonstrated that the improvement in mucociliary function was higher in the EM group than in the intranasal steroid group. In current study, reduction of mucus production after treatment of EM was significantly stronger than Dex. Disruption of epithelial barrier junction can enhance the infiltration of pathogens or allergens into the submucosal area, stimulating airway inflammation^10^. Soyka et al. reported the phenomena of lowered tight junction protein expressions in the epithelia of sinonasal tissues taken from patients with CRS^34^. Wise et al. reported e-cadherin expression decreased with IL-4 exposure to sinonasal epithelium^35^. In our current study, we found a tendency of reduced e-cadherin expression in CRS subjects and mice. However, the effect of EM on restoring nasal epithelial barrier dysfunction was not evident in our CRS mice.

Cytokines and chemokines are key regulators of the inflammatory responses. Previous studies of macrolides on CRS patients revealed no consistent effects on cytokine productions. Wallwork et al.^31^ conducted a trial of roxithromycin on CRS patients and found a reduction of IL-8 in nasal lavage after treatment. The anti-inflammatory function of macrolides is believed coming from both antibiotic and non-antibacterial effect. Macrolide treatment efficacy has shown in patients infected with pathogens insensitive to macrolides like pseudomonas aeruginosa^8^. Sadamatsu et al.^36^ treated asthmatic mice with a macrolide EM900 (a derivative of EM without antibacterial effects) and found significantly lower levels of IL-5, IL-13, RANTES, and IL-17A in their bronchoalveolar lavage. EM 900 also demonstrated inhibitory effect on IL-8 on culture human nasal epithelial cells in another in vitro study^37^. Pukhyalsky et al.^38^ studied patients with cystic fibrosis after prolonged treatments of clarithromycin. They found significant improvements of lung function and a switch from Th-2 to Th-1 cytokines in their blood and sputum. Interestingly, Park et al.^39^ found in patients with panbronchiolitis, a shift from Th-1 to Th-2 cytokine production in bronchoalveolar lavage fluid after long-term treatment with EM. The Th-1/Th-2 cytokines switches observed in the two human studies could reflect an immune-modulatory effect of macrolides. In current study, we also found the immunomodulatory effect in the EM-treated mice.

Clinical studies of macrolides on CRS involved application of various drugs and at different dosages. But the optimal drug, dosage, or duration are not currently known. Subgroup analyses of Seresirikachorn’s meta-analysis showed the results favor patients receiving macrolides at a half of regular antibiotics dose, and favor macrolide treatment for a duration of 24 weeks instead of shorter periods^22^. In our current study, we found that medium and high doses (7.5 and 75 mg/kg) worked well for treating of CRS. No dose–response could be with only 3 doses were used in our study. A meta-analysis reported that adding a macrolide to an intranasal steroid may achieve better results than using steroid alone to treat CRS^40^. Combined two drugs could be considered for patients unresponsive to monotherapy. In conclusion, we identified that comorbid AR exacerbates CRS severity. Our mice experiments showed Dex and EM treatments effects on CRS acted through somewhat different mechanisms.

## Supplementary Information

Supplementary Information 1.

Supplementary Information 2.

## References

[1] Fokkens WJ (2020). European position paper on rhinosinusitis and nasal polyps 2020. Rhinology.

[2] Orlandi RR (2021). International consensus statement on allergy and rhinology: rhinosinusitis 2021. Int. Forum Allergy Rhinol..

[3] Houser SM, Keen KJ (2008). The role of allergy and smoking in chronic rhinosinusitis and polyposis. Laryngoscope.

[4] Kirtsreesakul V, Ruttanaphol S (2008). The relationship between allergy and rhinosinusitis. Rhinology.

[5] Orlandi RR (2016). International consensus statement on allergy and rhinology: rhinosinusitis. Int. Forum Allergy Rhinol..

[6] Wilson KF, McMains KC, Orlandi RR (2014). The association between allergy and chronic rhinosinusitis with and without nasal polyps: an evidence-based review with recommendations. Int. Forum Allergy Rhinol..

[7] Ganesan S, Comstock AT, Sajjan US (2013). Barrier function of airway tract epithelium. Tissue Barriers.

[8] Shimizu T, Shimizu S, Hattori R, Gabazza EC, Majima Y (2003). In vivo and in vitro effects of macrolide antibiotics on mucus secretion in airway epithelial cells. Am. J. Respir. Crit. Care Med..

[9] Tong J, Gu Q (2020). Expression and clinical significance of mucin gene in chronic rhinosinusitis. Curr. Allergy Asthma Rep..

[10] Fukuoka A, Yoshimoto T (2018). Barrier dysfunction in the nasal allergy. Allergol. Int. Off. J. Jpn. Soc. Allergol..

[11] Steelant B (2016). Impaired barrier function in patients with house dust mite-induced allergic rhinitis is accompanied by decreased occludin and zonula occludens-1 expression. J. Allergy Clin. Immunol..

[12] Antunes MB, Gudis DA, Cohen NA (2009). Epithelium, cilia, and mucus: their importance in chronic rhinosinusitis. Immunol. Allergy Clin. N. Am..

[13] Stevens WW, Schleimer RP, Kern RC (2016). Chronic rhinosinusitis with nasal polyps. J. Allergy Clin. Immunol. Practice.

[14] Stevens WW, Lee RJ, Schleimer RP, Cohen NA (2015). Chronic rhinosinusitis pathogenesis. J. Allergy Clin. Immunol..

[15] Van Crombruggen K, Zhang N, Gevaert P, Tomassen P, Bachert C (2011). Pathogenesis of chronic rhinosinusitis: inflammation. J. Allergy Clin. Immunol..

[16] Tomassen P (2016). Inflammatory endotypes of chronic rhinosinusitis based on cluster analysis of biomarkers. J. Aallergy Clin. Immunol..

[17] Bachert C, Akdis CA (2016). Phenotypes and emerging endotypes of chronic rhinosinusitis. J. Allergy Clin. Immunol. Practice.

[18] Bachert C, Zhang N, Hellings PW, Bousquet J (2018). Endotype-driven care pathways in patients with chronic rhinosinusitis. J. Allergy Clin. Immunol..

[19] Lin X, Lu J, Yang M, Dong BR, Wu HM (2015). Macrolides for diffuse panbronchiolitis. Cochrane Database Syst. Rev..

[20] Kanoh S, Rubin BK (2010). Mechanisms of action and clinical application of macrolides as immunomodulatory medications. Clin. Microbiol. Rev..

[21] Cervin A (2001). The anti-inflammatory effect of erythromycin and its derivatives, with special reference to nasal polyposis and chronic sinusitis. Acta Otolaryngol..

[22] Seresirikachorn K (2019). Factors of success of low-dose macrolides in chronic sinusitis: systematic review and meta-analysis. Laryngoscope.

[23] Perić A, Vojvodić D, Matković-Jožin S (2012). Effect of long-term, low-dose clarithromycin on T helper 2 cytokines, eosinophilic cationic protein and the 'regulated on activation, normal T cell expressed and secreted' chemokine in the nasal secretions of patients with nasal polyposis. J. Laryngol. Otol..

[24] Lund VJ, Kennedy DW (1997). Staging for rhinosinusitis. Otolaryngol. Head Neck Surg..

[25] Liang KL (2013). Upper airway inflammation exacerbates bronchial hyperreactivity in mouse models of rhinosinusitis and allergic asthma. Int. Forum Allergy Rhinol..

[26] Al-Sayed AA, Agu RU, Massoud E (2017). Models for the study of nasal and sinus physiology in health and disease: a review of the literature. Laryngosc. Investig. Otolaryngol..

[27] Vlaminck S (2014). The importance of local eosinophilia in the surgical outcome of chronic rhinosinusitis: a 3-year prospective observational study. Am. J. Rhinol. Allergy.

[28] Kilkenny C, Browne WJ, Cuthill IC, Emerson M, Altman DG (2010). Improving bioscience research reporting: the ARRIVE guidelines for reporting animal research. PLoS Biol..

[29] Xiang R (2019). Different effects of allergic rhinitis on nasal mucosa remodeling in chronic rhinosinusitis with and without nasal polyps. Eur. Arch. Otorhinolaryngol. Off. J. Eur. Fed. Otorhinolaryngol. Soc..

[30] Hellings PW (2017). Positioning the principles of precision medicine in care pathways for allergic rhinitis and chronic rhinosinusitis—a EUFOREA-ARIA-EPOS-AIRWAYS ICP statement. Allergy.

[31] Wallwork B, Coman W, Mackay-Sim A, Greiff L, Cervin A (2006). A double-blind, randomized, placebo-controlled trial of macrolide in the treatment of chronic rhinosinusitis. Laryngoscope.

[32] Haxel BR (2015). Controlled trial for long-term low-dose erythromycin after sinus surgery for chronic rhinosinusitis. Laryngoscope.

[33] Wu SH, Hsu SH, Liang KL, Jiang RS (2019). The effects of erythromycin towards the treatment of persistent rhinosinusitis after functional endoscopic sinus surgery: a randomized, active comparator-controlled study. J. Chin. Med. Assoc..

[34] Soyka MB (2012). Defective epithelial barrier in chronic rhinosinusitis: the regulation of tight junctions by IFN-gamma and IL-4. J. Allergy Clin. Immunol..

[35] Wise SK (2014). Interleukin-4 and interleukin-13 compromise the sinonasal epithelial barrier and perturb intercellular junction protein expression. Int. Forum Allergy Rhinol..

[36] Sadamatsu H (2020). The non-antibiotic macrolide EM900 attenuates HDM and poly(I:C)-induced airway inflammation with inhibition of macrophages in a mouse model. Inflam. Res. Off. J. Eur. Histamine Res. Soc..

[37] Wakayama N (2018). Anti-inflammatory effects of EM900 on cultured human nasal epithelial cells. J. Nippon Med. Sch..

[38] Pukhalsky AL (2004). Anti-inflammatory and immunomodulating effects of clarithromycin in patients with cystic fibrosis lung disease. Mediators Inflamm..

[39] Park SJ, Lee YC, Rhee YK, Lee HB (2004). The effect of long-term treatment with erythromycin on Th1 and Th2 cytokines in diffuse panbronchiolitis. Biochem. Biophys. Res. Commun..

[40] Huang Z, Zhou B (2019). Clarithromycin for the treatment of adult chronic rhinosinusitis: a systematic review and meta-analysis. Int. Forum Allergy Rhinol..

