# Supplementary material for: Erythromycin reduces nasal inflammation by inhibiting immunoglobulin production, attenuating mucus secretion, and modulating cytokine expression
Source: Sci Rep. 2021 Nov 5;11:21737. doi: 10.1038/s41598-021-01192-8 (PMC8571277; doi:10.1038/s41598-021-01192-8)

Supplementary Fig.1: Full-length western blotting shown in Fig. 2C right.

MUC5AC


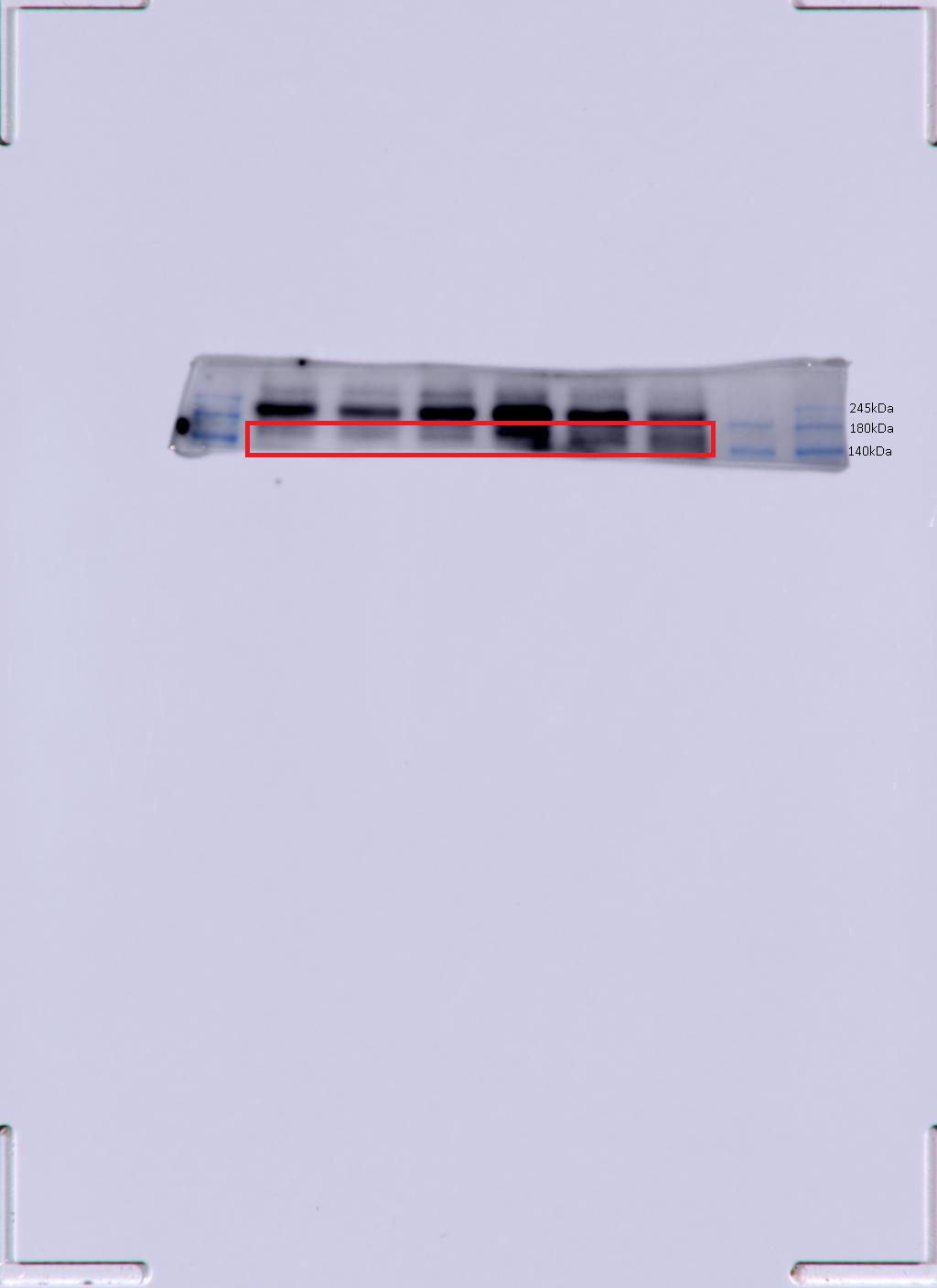


E-cadherin


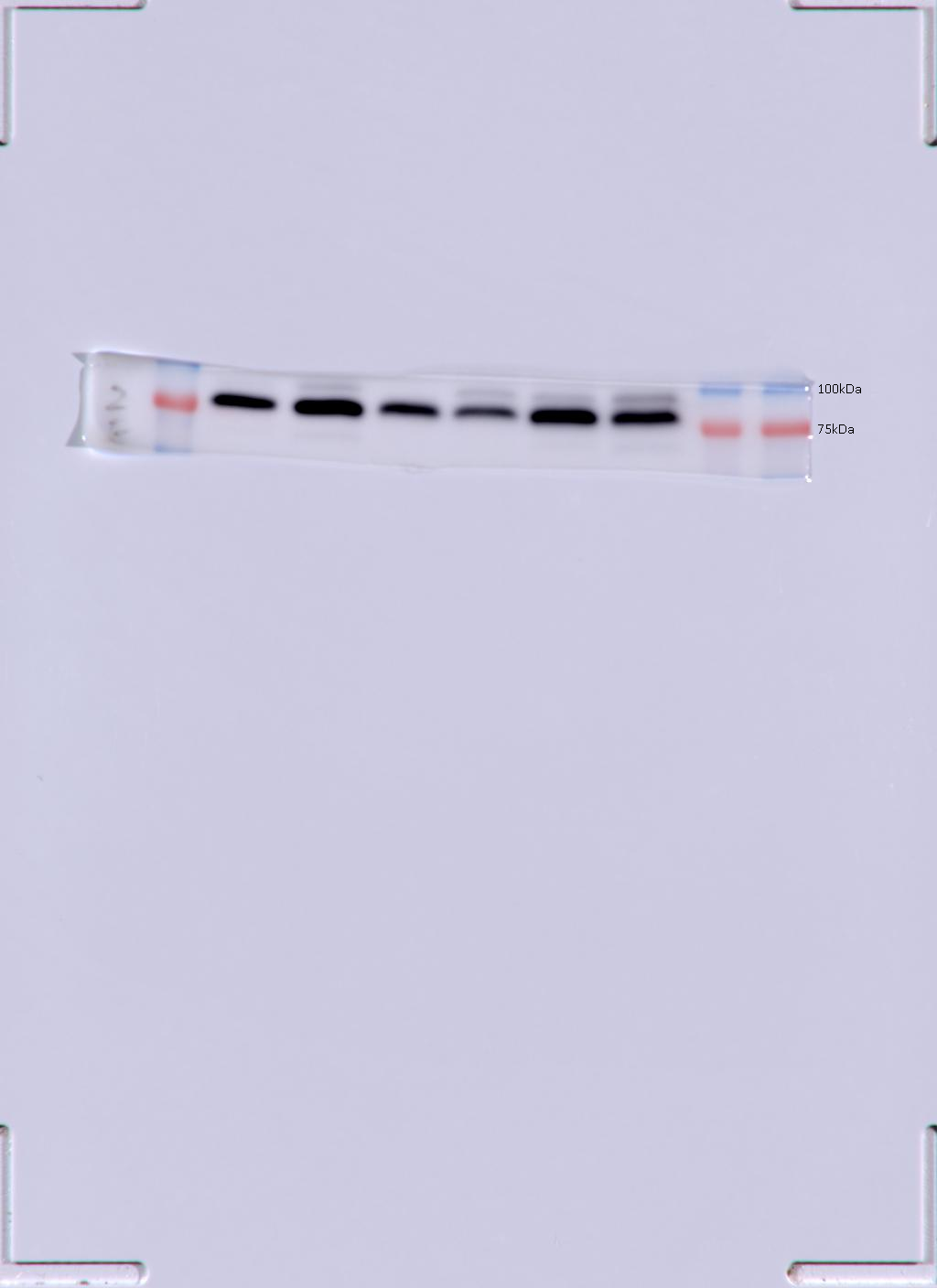


Claudin-1


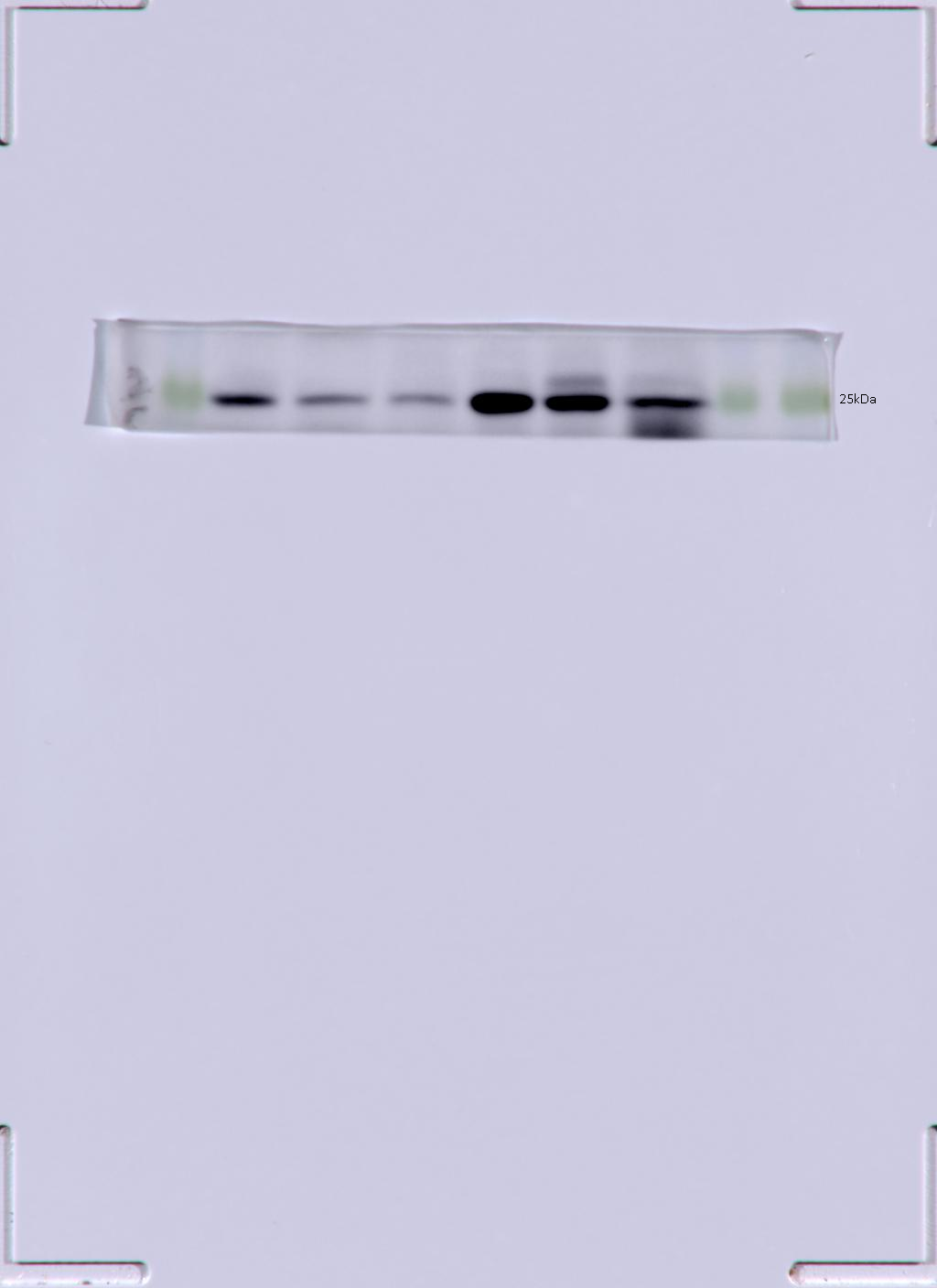


GAPDH


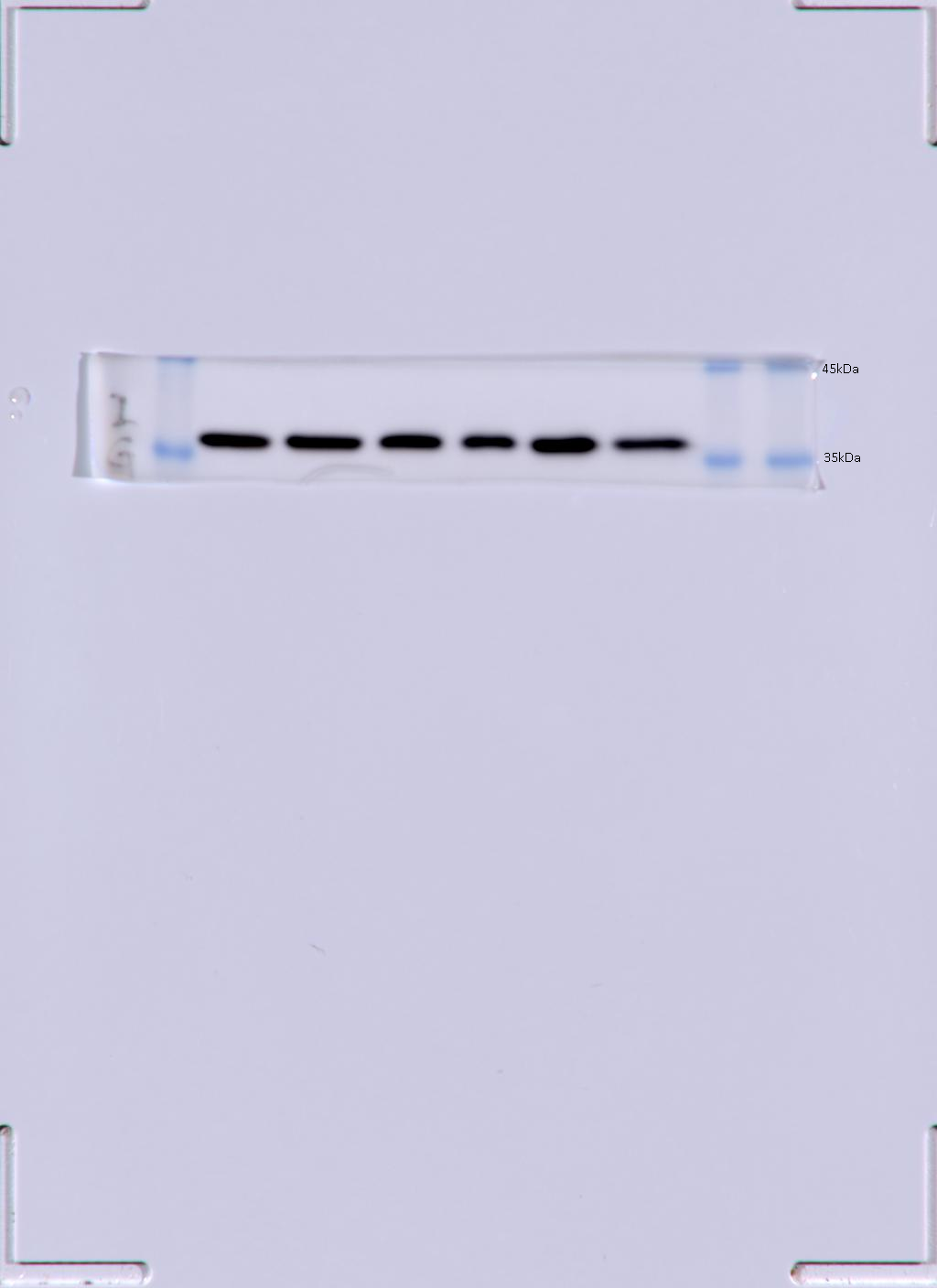


Supplementary Fig. 2: Full-length western blotting shown in Fig. 2C left.

MUC5AC


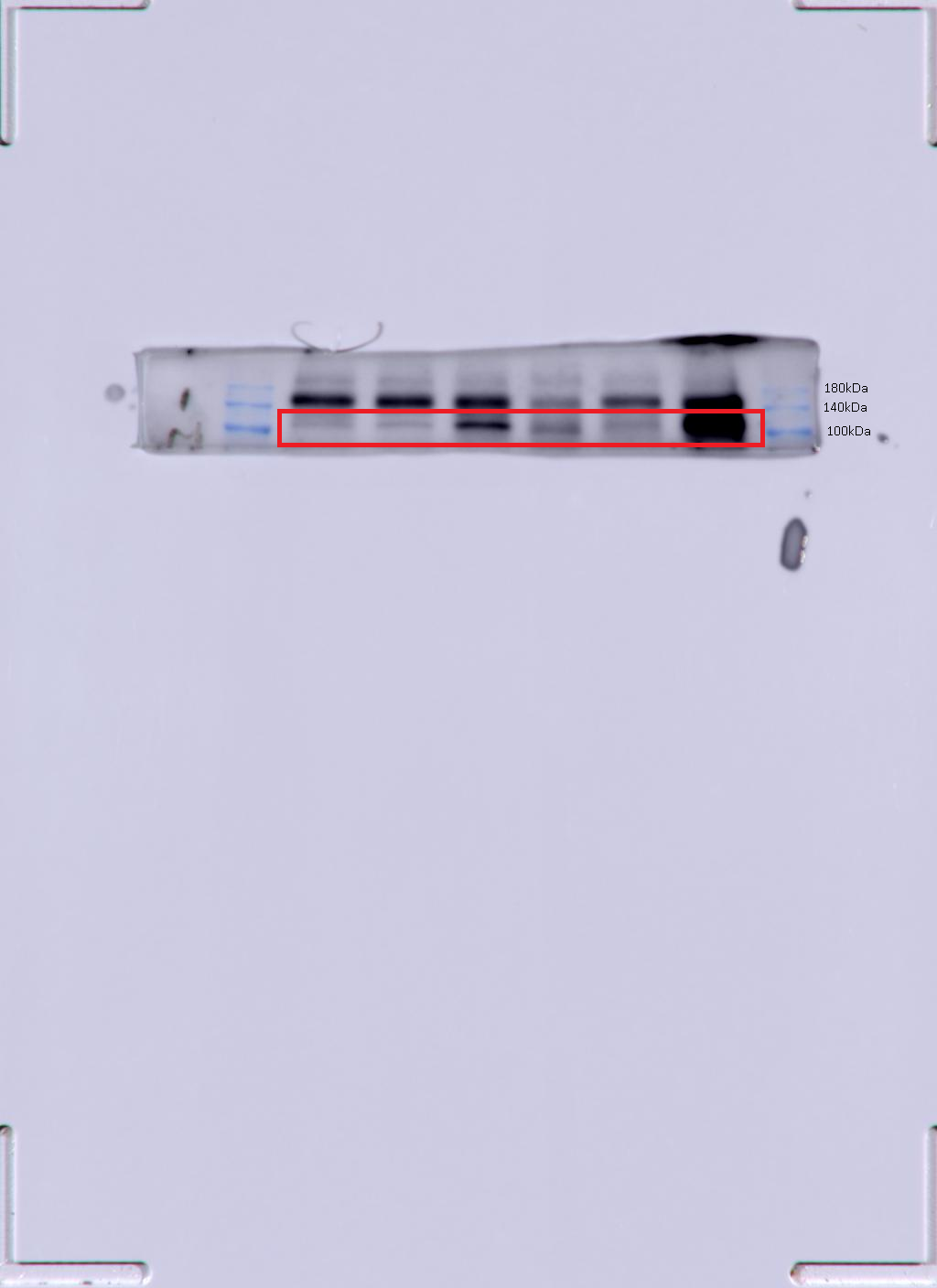


E-cadherin


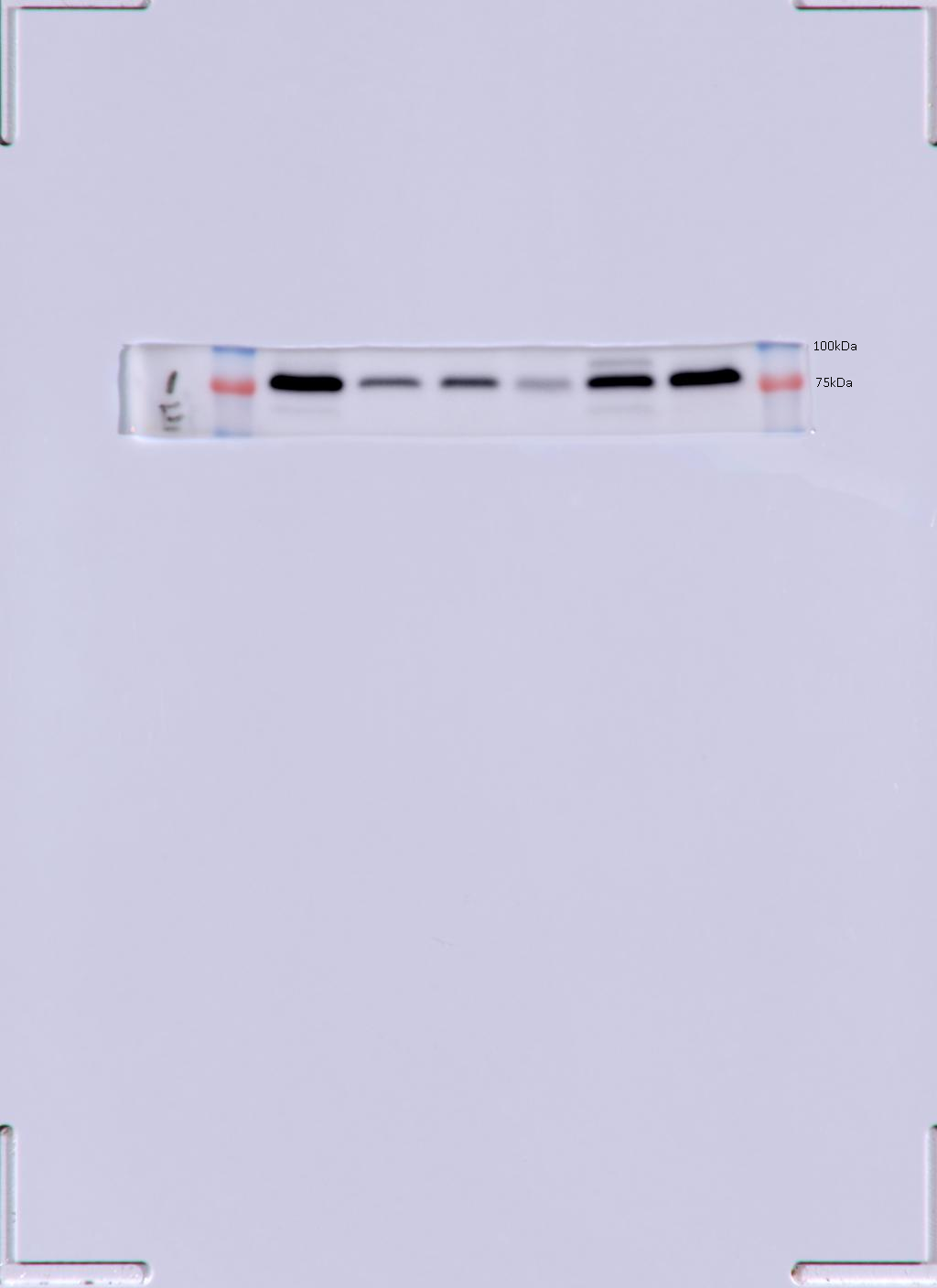


Claudin-1


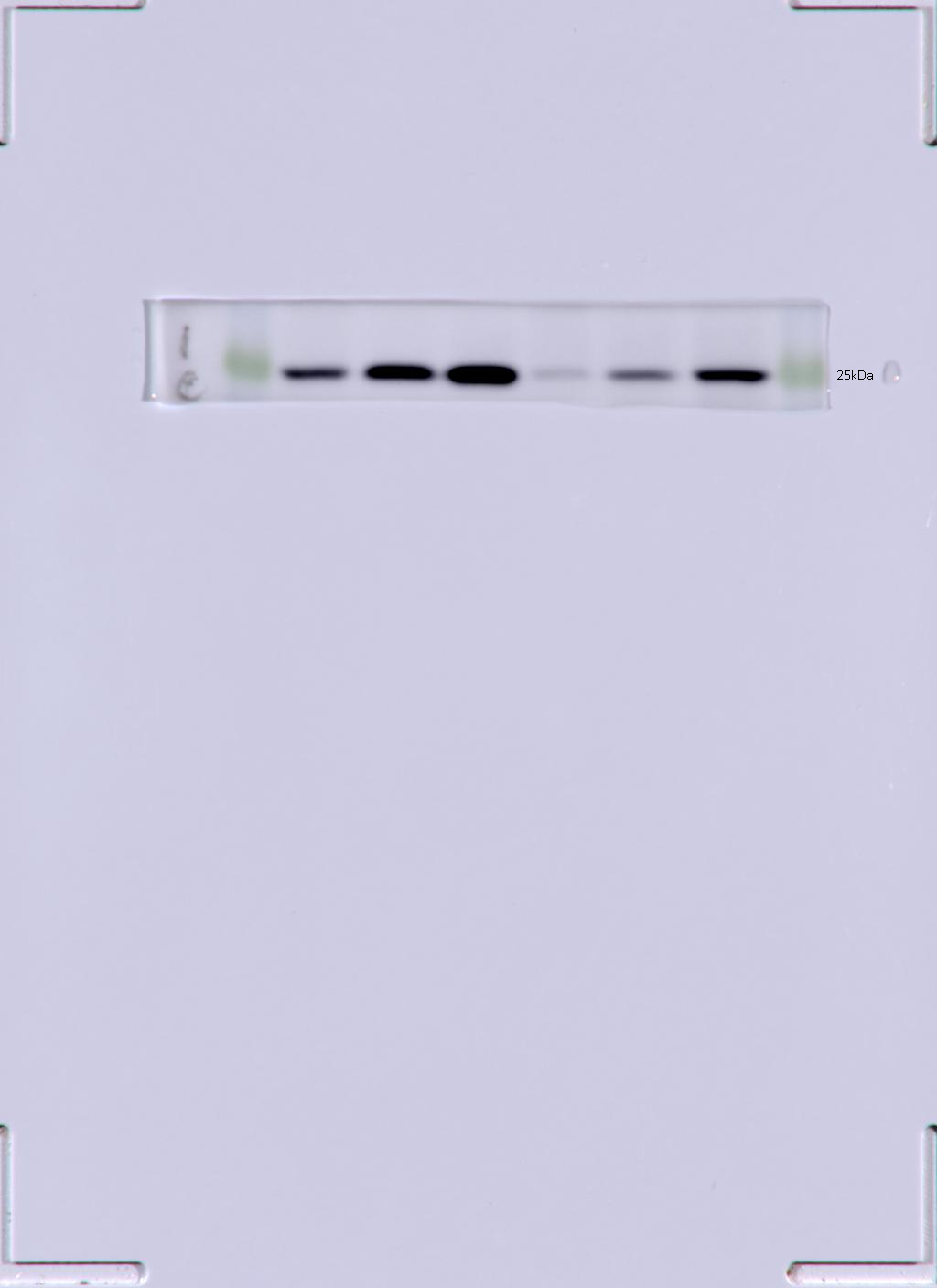


GAPDH


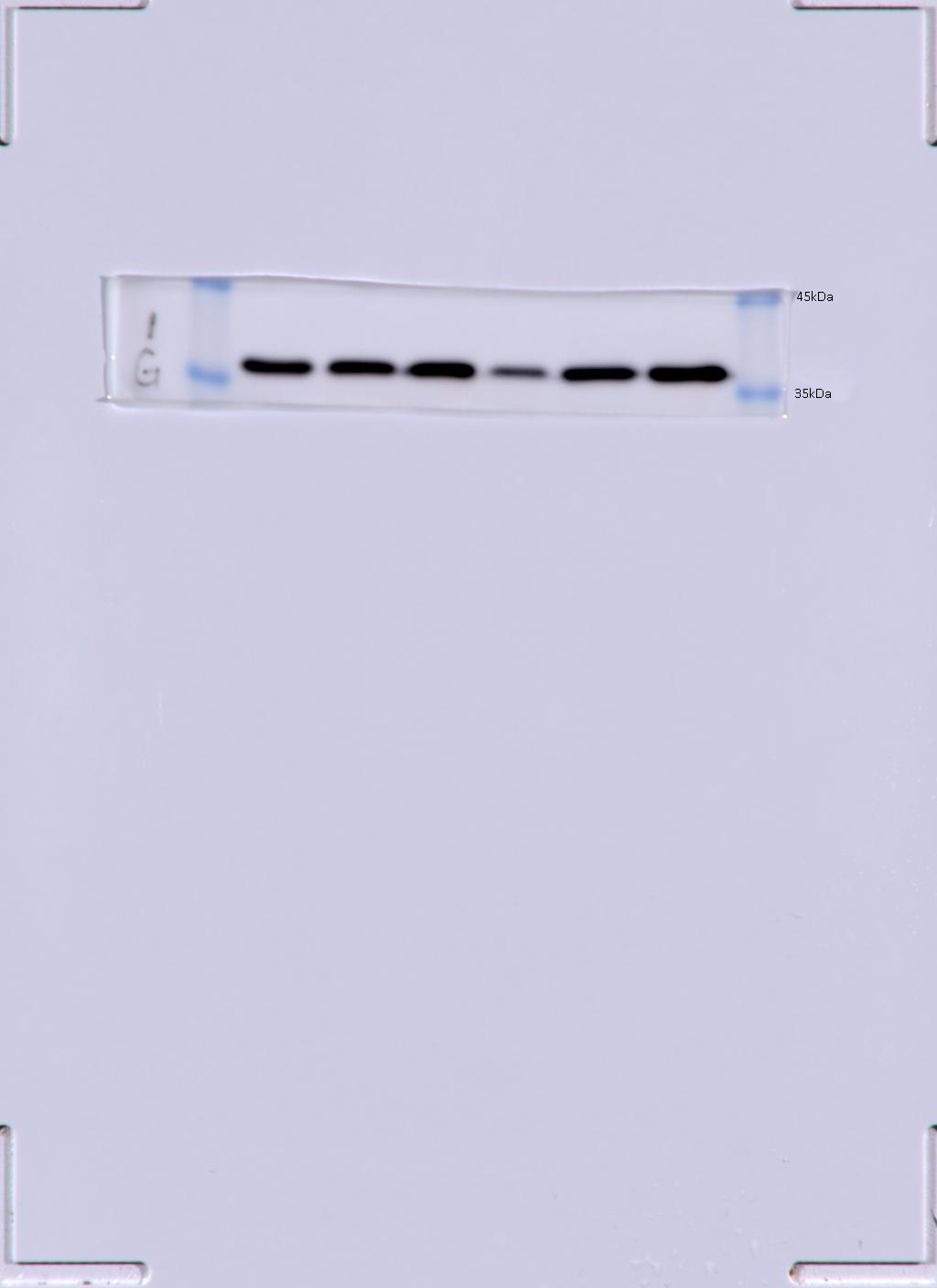


Supplementary Fig. 3: Full-length western blotting shown in Fig. 6B right.

MUC5AC


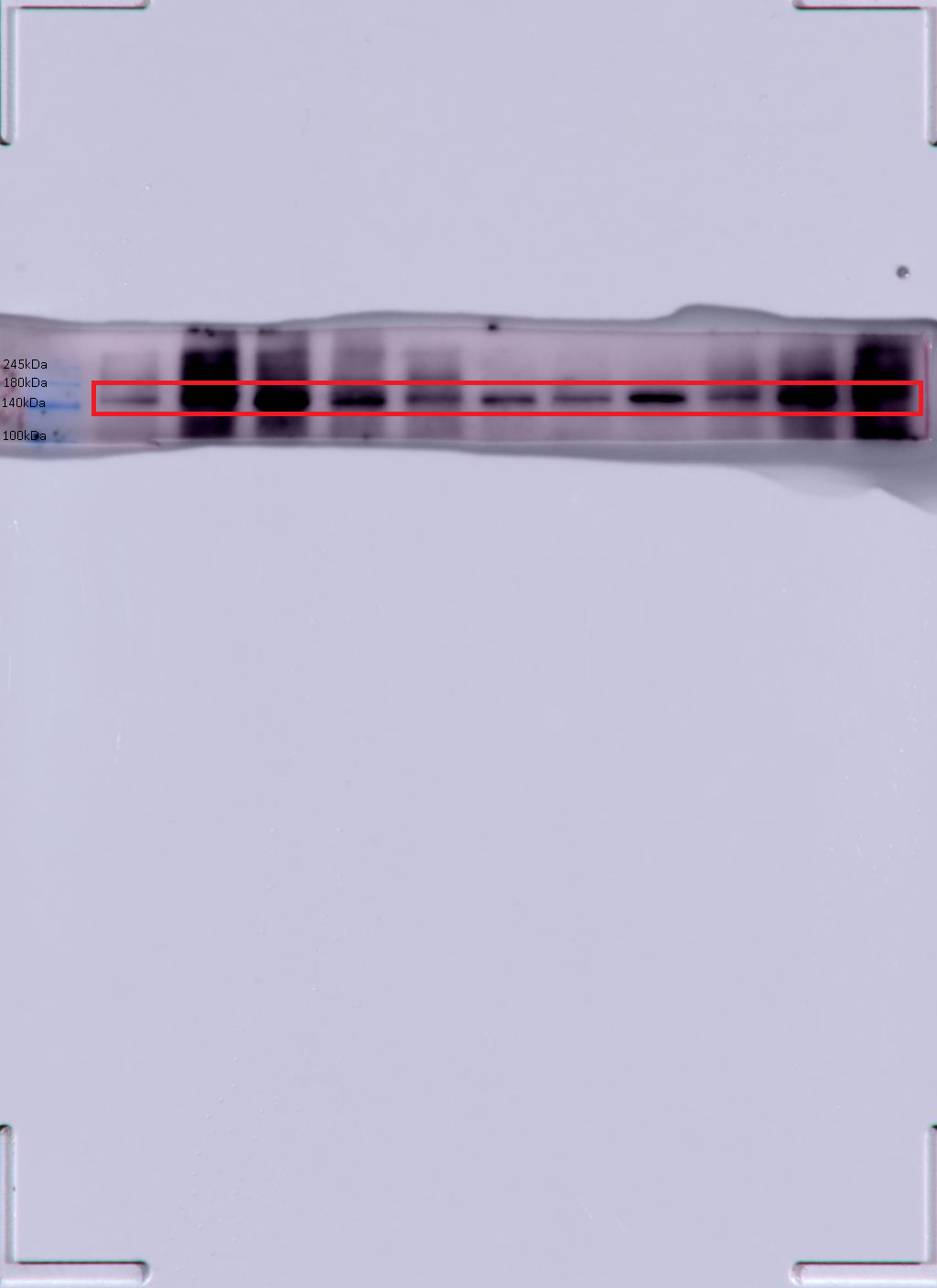


E-cadherin


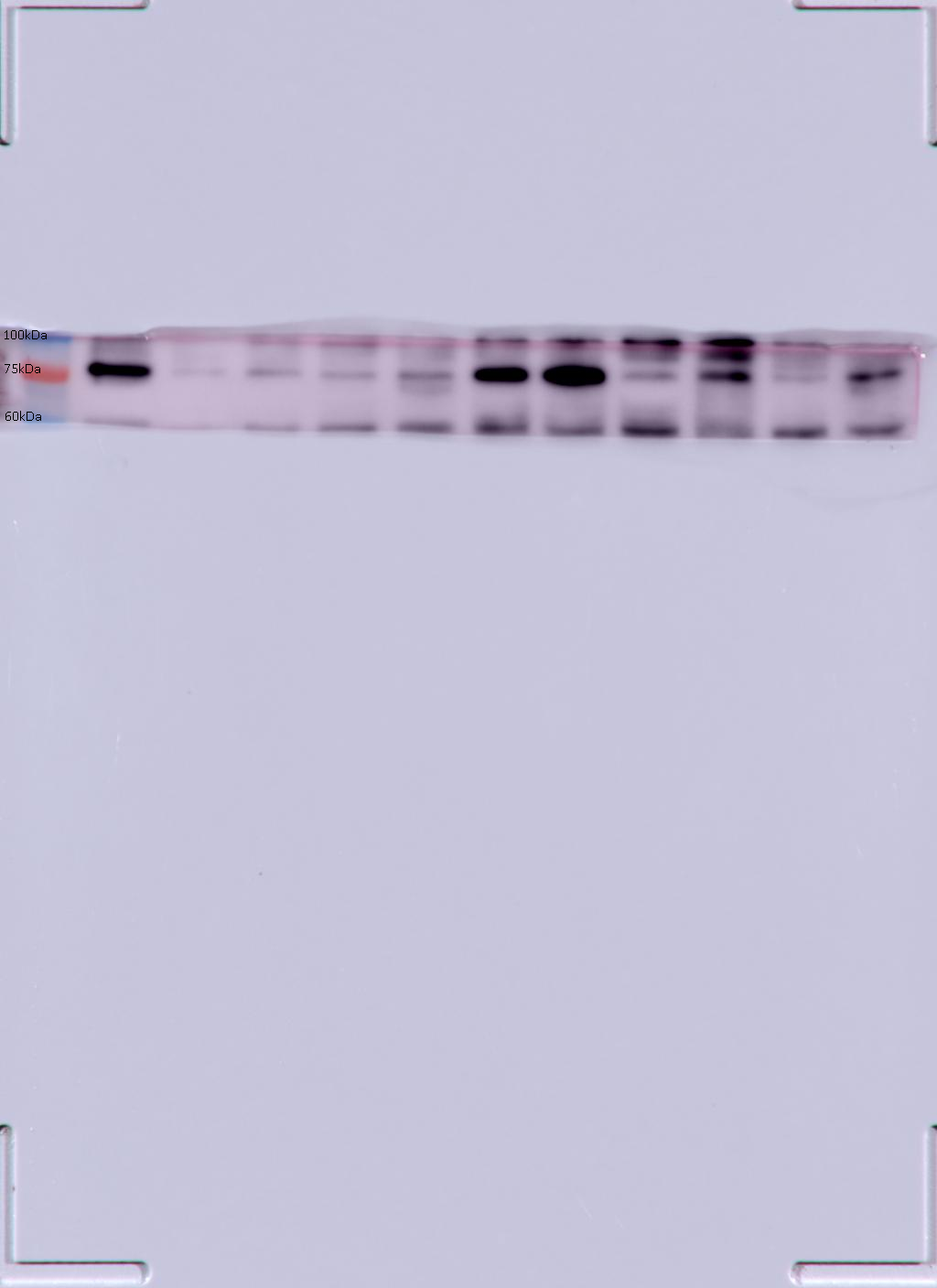


Claudin-1


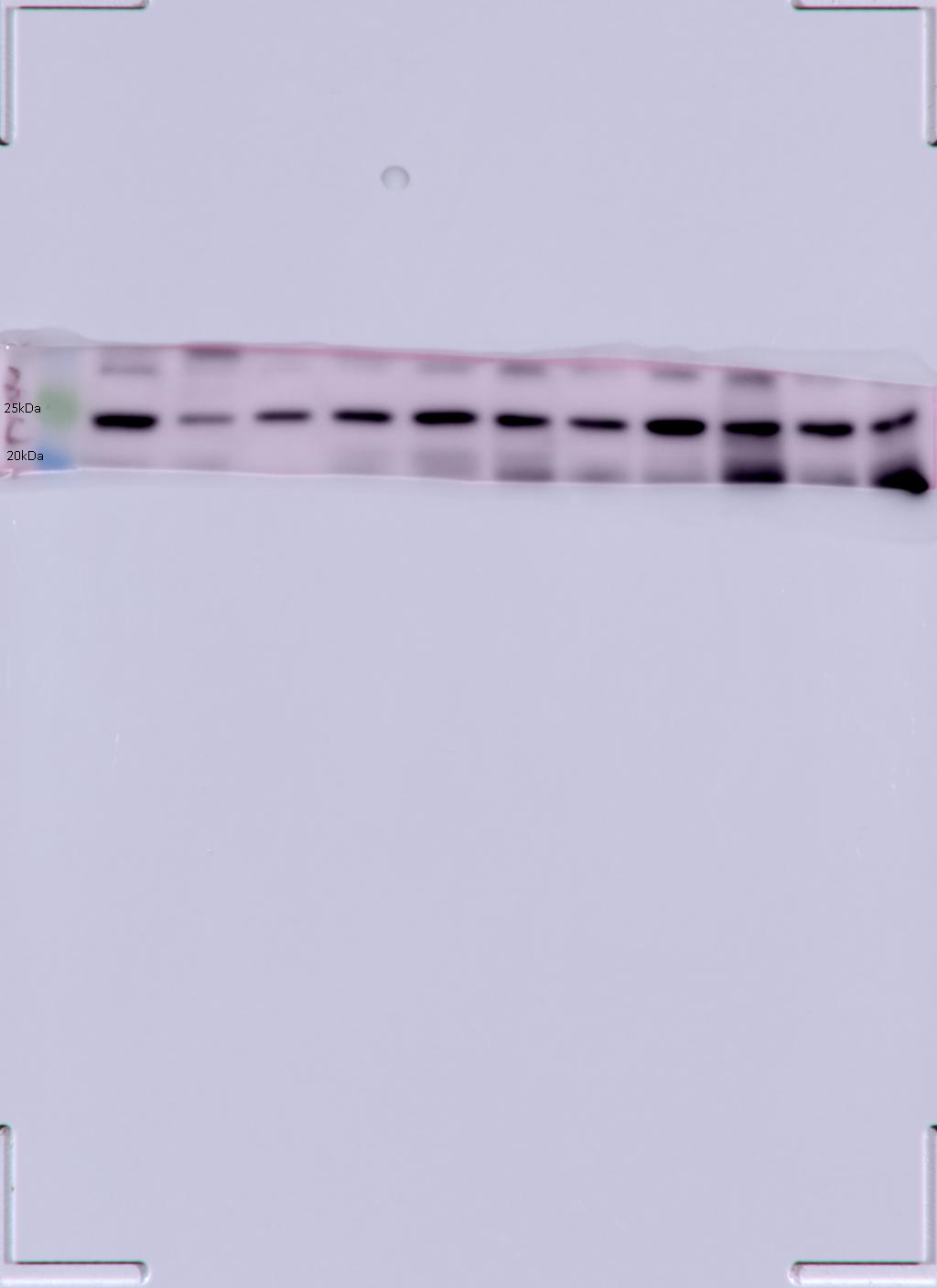


GAPDH


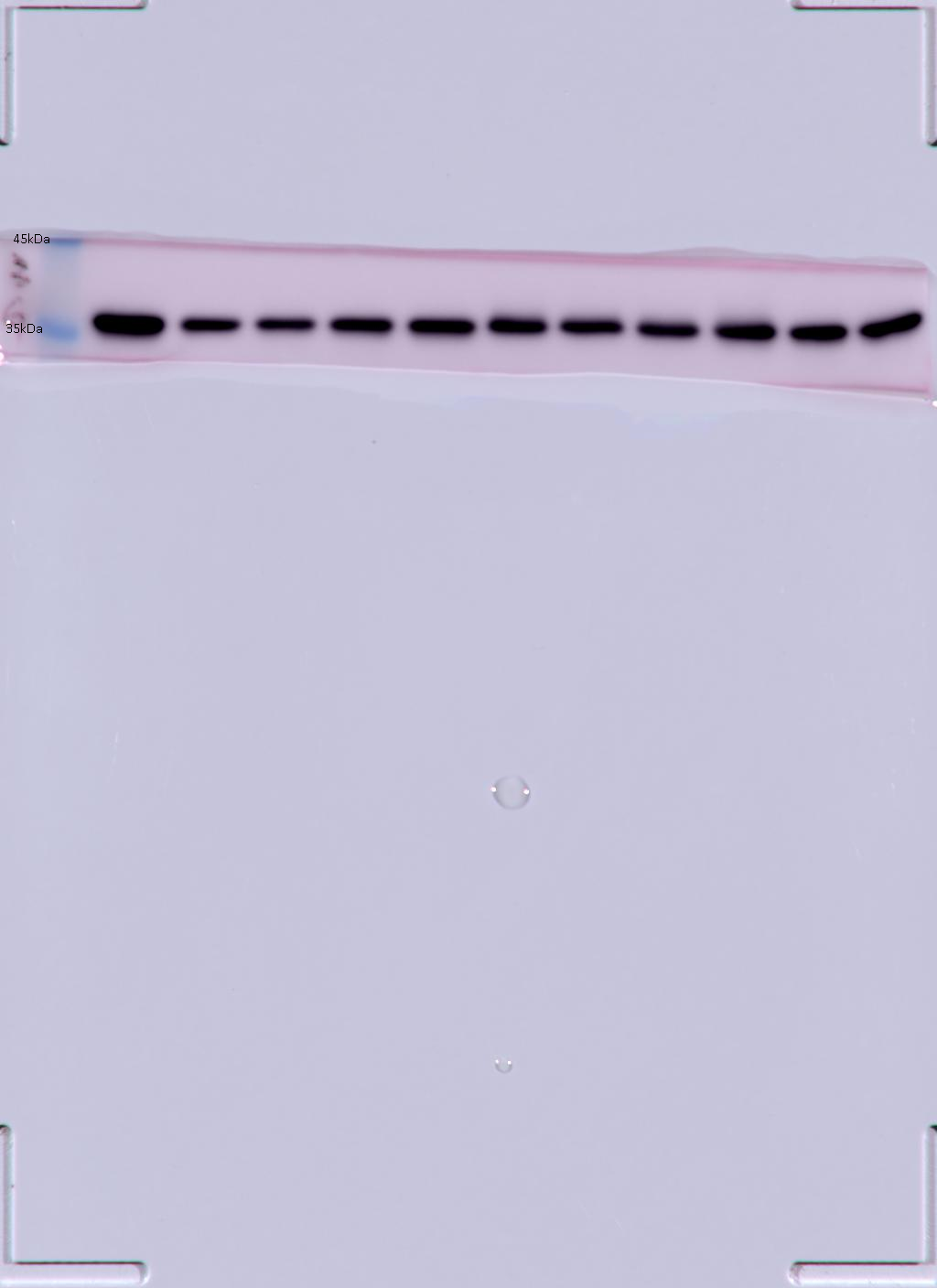


Supplementary Fig. 4: Full-length western blotting shown in Fig. 6B left.

MUC5AC


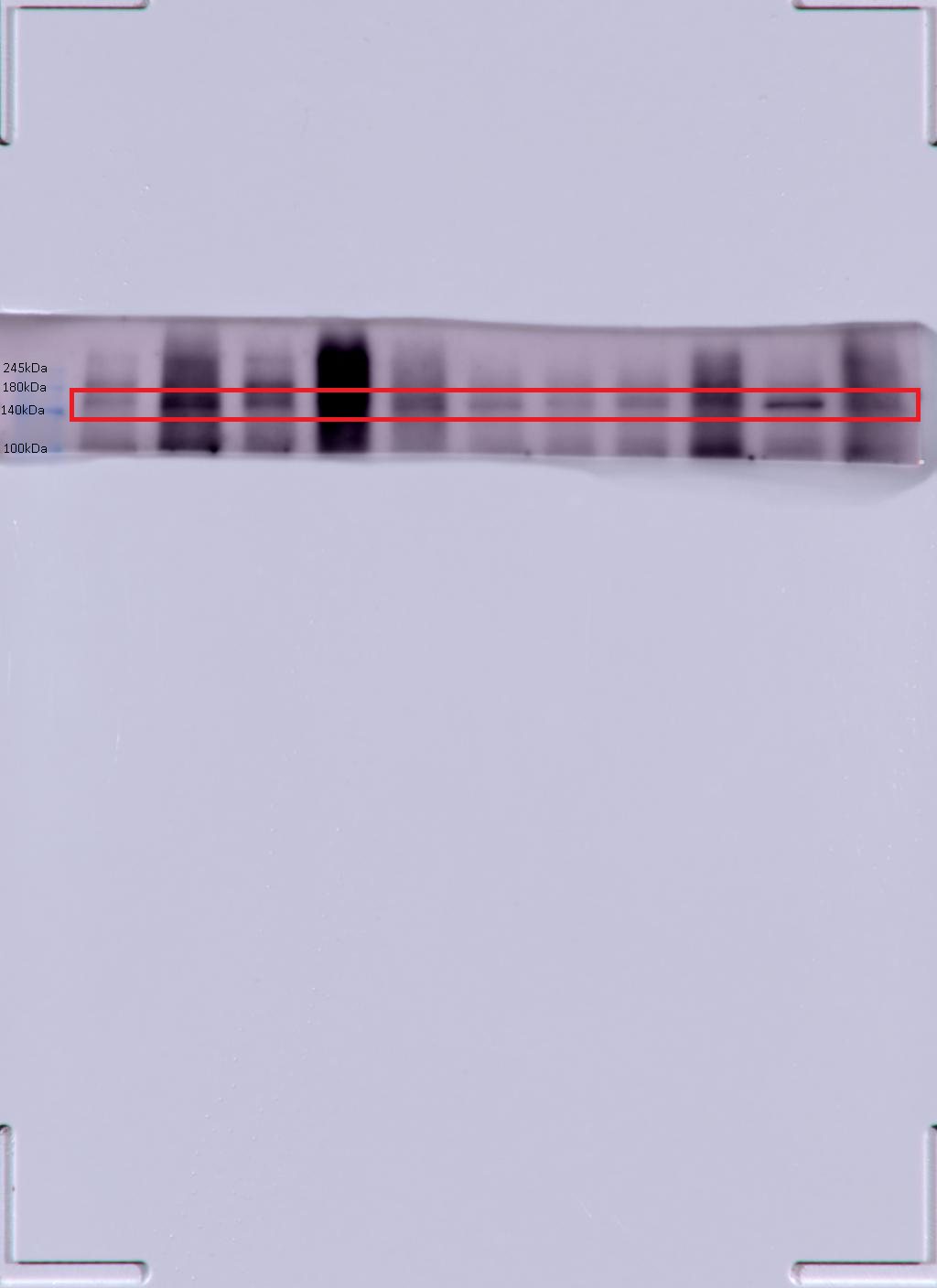


E-cadherin


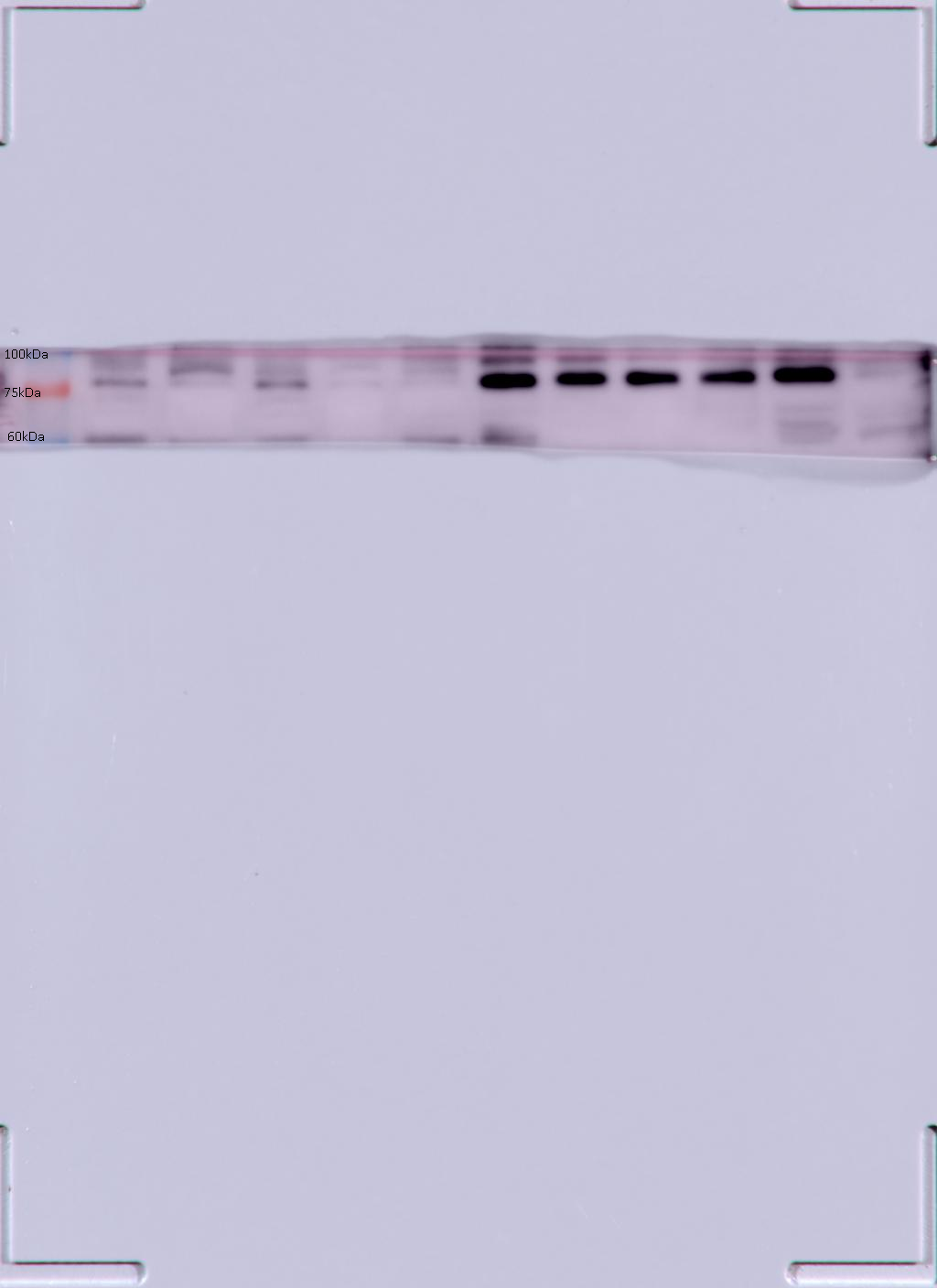


Claudin-1


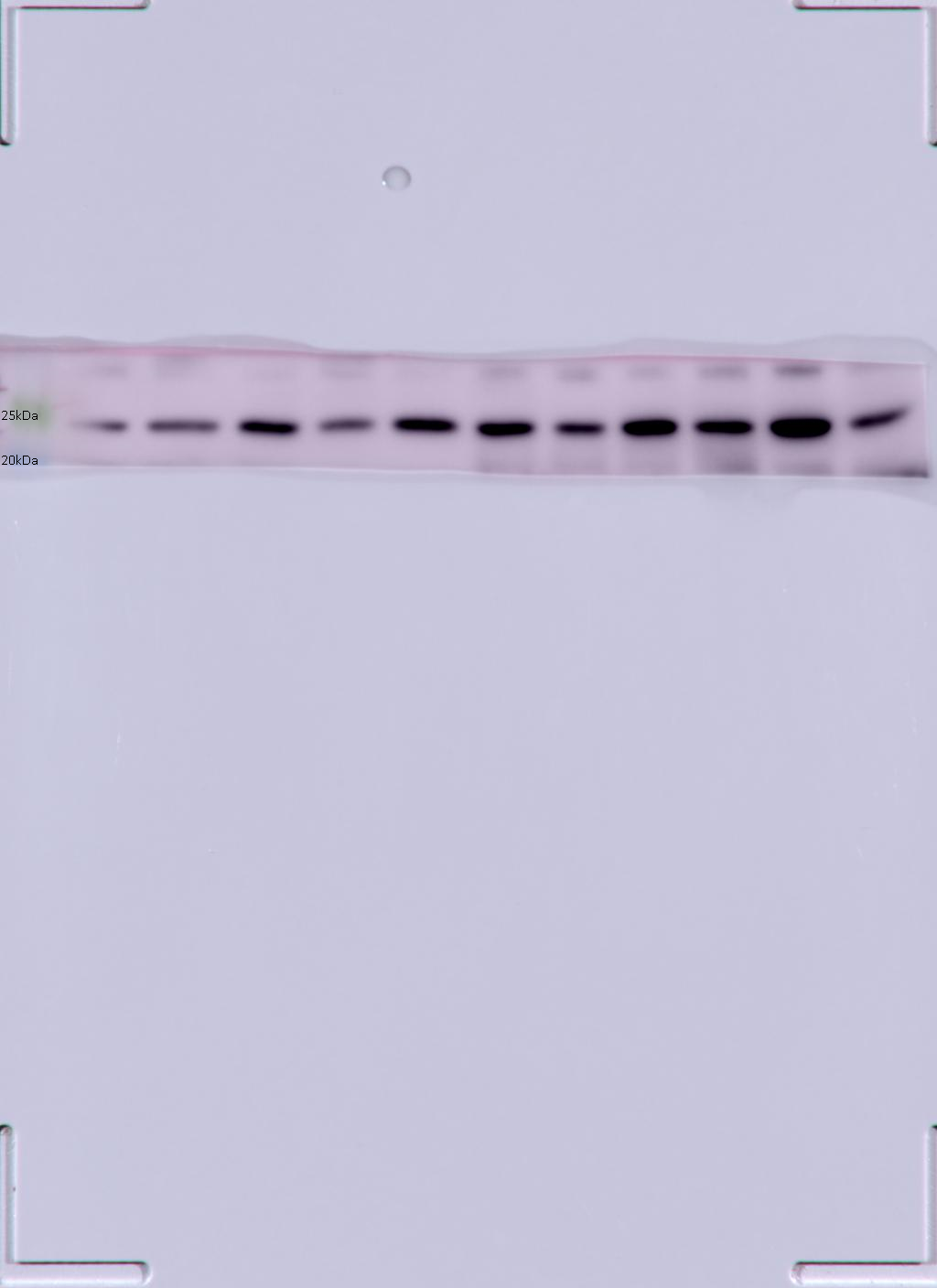


GAPDH


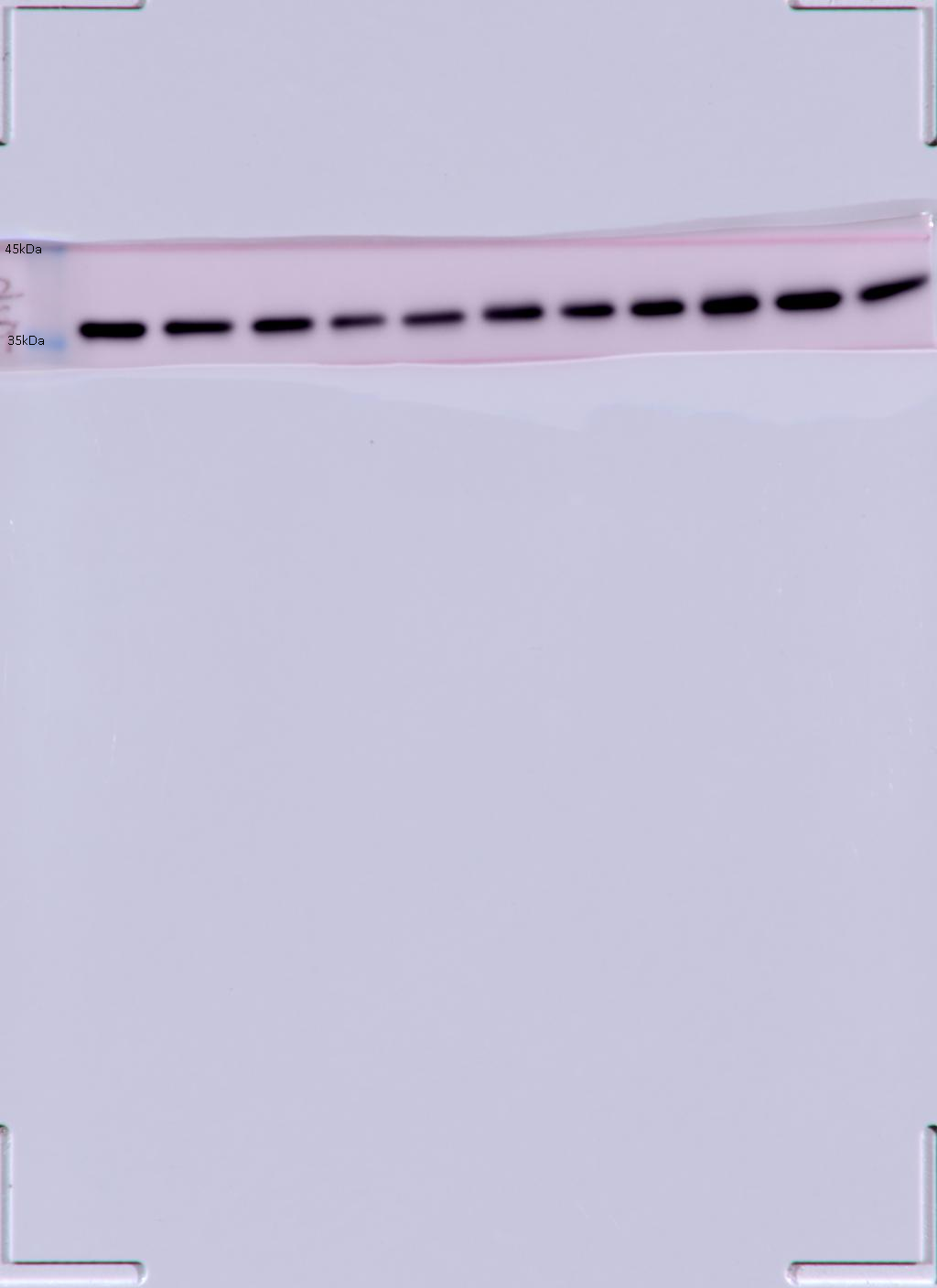

Supplement: Supplementary file 1 — Supplementary Information 1. [file 41598_2021_1192_MOESM1_ESM.docx]
