# Supplementary material for: Erythromycin reduces nasal inflammation by inhibiting immunoglobulin production, attenuating mucus secretion, and modulating cytokine expression
Source: Sci Rep. 2021 Nov 5;11:21737. doi: 10.1038/s41598-021-01192-8 (PMC8571277; doi:10.1038/s41598-021-01192-8)
Supplement: Supplementary file 2 — Supplementary Information 2. [file 41598_2021_1192_MOESM2_ESM.docx]

1. **Human study**

Supplementary Table 1. Cytokine levels of nasal mucosa in chronic rhinosinusitis patients with or without allergic rhinitis.

| Group | Control (N=15) | CRSwoAR (N=19) | CRSwAR (N=19) | P value |
| --- | --- | --- | --- | --- |
| IL-4  (mean ± SEM) | 0.90±0.16 | 1.90±0.35 | 2.76±0.45 | 0.0040*^a^ |
| Post hoc^b^ | CRSwoAR vs Control, P=0.2141; CRSwAR vs. Control, P = 0.0027*; CRSwoAR vs. CRSwAR, P =0.3169 | | | |
| IL-5 | 10.23±4.04 | 29.07±6.01 | 51.04±7.56 | 0.0006*^a^ |
| Post hoc^b^ | CRSwoAR vs Control, P=0.0911; CRSwAR vs. Control, P=0.0004*; CRSwoAR vs. CRSwAR, P=0.2036 | | | |
| IL-10 | 2.75±0.77 | 6.21±1.20 | 8.69±1.00 | 0.0023*^a^ |
| Post hoc^b^ | CRSwoAR vs Control, P = 0.2488; CRSwAR vs. Control, P=0.0015*; CRSwoAR vs. CRSwAR, P =0.2639 | | | |
| IFN-𝛾 | 10.57±1.05 | 29.50±8.49 | 33.01±5.71 | 0.0008*^a^ |
| Post hoc^b^ | CRSwoAR vs Control, P =0.0473*; CRSwAR vs. Control, P=0.0005*; CRSwoAR vs. CRSwAR, P =0.4593 | | | |
| IL-6 | 96.40±26.3 | 141.5±62.86 | 150.3±38.26 | 0.2595^a^ |
| Post hoc^b^ | CRSwoAR vs Control, CRSwAR vs. Control, both P>0.9999; CRSwoAR vs. CRSwAR, P =0.3105 | | | |
| IL-17A | 18.23±2.45 | 29.87±4.99 | 40.85±6.57 | 0.0257*^a^ |
| Post hoc^b^ | CRSwoAR vs Control, P =0.4037; CRSwAR vs. Control, P =0.0205*; CRSwoAR vs. CRSwAR, P =0.5943 | | | |

CRSwoAR = chronic rhinosinusitis without allergic rhinitis; CRSwAR = chronic rhinosinusitis with allergic rhinitis; ^a^ Kruskal-Wallis test; ^b^ Dunn’s multiple comparisons test; P<0.05.

Supplementary Table 2. Relative protein expression of nasal mucosa in chronic rhinosinusitis patients with or without allergic rhinitis.

| Group | Control (N=12) | CRSwoAR (N=20) | CRSwAR (N=18) | P value |  |
| --- | --- | --- | --- | --- | --- |
| MUC5AC  (mean ± SEM) | 0.39±0.06 | 0.70±0.11 | 0.58±0.10 | 0.1899^a^ |  |
| Post hoc^b^ | CRSwoAR vs Control, P=0.2056; CRSwAR vs. Control, P = 0.7243; CRSwoAR vs. CRSwAR, P >0.9999 | | | | |
| E-cadherin | 1.16±0.18 | 0.91±0.08 | 1.05±1.11 | 0.3014^a^ |  |
| Post hoc^b^ | CRSwoAR vs Control, P=0.5418; CRSwoAR vs. control, P >0.9999; CRSwAR vs. CRSwAR, P =0.6121 | | | | |
| Claudin-1 | 0.74±0.32 | 0.64±0.14 | 0.55±0.13 | 0.8877^a^ |  |
| Post hoc^b^ | CRSwoAR vs Control, CRSwAR vs. Control, CRSwoAR vs. CRSwAR, all P>0.9999 | | | | |

CRSwoAR = chronic rhinosinusitis without allergic rhinitis; CRSwAR = chronic rhinosinusitis with allergic rhinitis; ^a^ Kruskal-Wallis test; ^b^ Dunn’s multiple comparisons test.

1. **Experimental mice study**

Supplementary Table 3. Epithelial eosinophil numbers/5 high power fields in experimental chronic rhinosinusitis mice with or without allergic rhinitis.

| Group | Control | | CRSwoAR | | CRSwAR | | P value | |  |
| --- | --- | --- | --- | --- | --- | --- | --- | --- | --- |
| Eos count | 1.33±0.67 | | 8.33±0.58 | | 18.33±2.19 | | 0.0036*^a^ | |  |
| Post hoc^b^ | CRSwoAR vs Control, P=0.5282; CRSwAR vs. Control, P = 0.0204*; CRSwoAR vs. CRSwAR, P =0.5282 | | | | | | | | |
| Group | Chronic rhinosinusitis without allergic rhinitis | | | | | | |  | |
| Treatment | - | Erythromycin | | | | Dexamethasone | |  | |
| Dose | - | 0.75 mg/kg | 7.5 mg/kg | 75 mg/kg | | 1mg/kg | | P value | |
| Eos count | 8.33±0.33 | 3.00±0.58 | 2.67±0.67 | 1.33±0.33 | | 2.00±1.00 | | 0.0200*^a^ | |
| Post hoc^b^ | CRSwoAR vs. EM 0.75, EM 0.75 vs. EM 7.5, EM 0.75 vs. EM75, EM0.75 vs. Dex, EM 7.5vs. EM75, EM 7.5 vs, Dex, EM 75 vs Dex, All P>0.9999, CRSwoAR vs. EM 7.5, P =0.9274; CRSwoAR vs. EM 75, P = 0.0439*; CRSwoAR vs. Dex, P = 0.1517* | | | | | | |  | |
| Group | Chronic rhinosinusitis with allergic rhinitis | | | | | | |  | |
| Treatment | - | Erythromycin | | | | Dexamethasone | |  | |
| Dose | - | 0.75 mg/kg | 7.5 mg/kg | 75 mg/kg | | 1mg/kg | | P value | |
| Eos count | 18.33±2.19 | 8.67±0.67 | 10.33±1.86 | 8.00±0.58 | | 0.67±0.33 | | 0.0017*^a^ | |
| Post hoc^b^ | CRSwAR vs, EM 0.75, CRSwAR vs. EM7.5, EM0.75 vs. EM 7.5, EM 0.75 vs. EM75, EM 7.5 vs, EM 75, EM 75 vs. Dex, all P>0.9999; CRSwAR vs EM75, P=0.4705; CRSwAR vs, Dex, P=0.0105*; EM 0.75 vs. Dex, P =0.9640; EM 7.5 vs. Dex, P =0.3769. | | | | | | |  | |

Control=non-chronic rhinosinusitis, non-allergic rhinitis mice; CRSwoAR =chronic rhinosinusitis without allergic rhinitis mice; CRSwAR = chronic rhinosinusitis with allergic rhinitis mice; ^a^ Kruskal-Wallis test; ^b^ Dunn’s multiple comparisons test; P<0.05.

Supplementary Table 4. Immunoglobulin levels of experimental chronic rhinosinusitis mice with or without allergic rhinitis.

| Group | Control (N=17) | CRSwoAR (N=19) | CRSwAR (N=26) | P value |  |
| --- | --- | --- | --- | --- | --- |
| Total IgE 𝚞g/ml  (mean ± SEM) | 0.24±0.82 | 1.29±0.30 | 1.29±0.3 | <0.0001*^a^ |  |
| Post hoc^b^ | CRSwoAR vs Control, P=0.4799; CRSwAR vs. Control, CRSwoAR vs. CRSwAR, both P <0.0001* | | | | |
| HDM IgE (O.D.) | 0.15±0.01 | 0.16±0.01 | 0.42±0.05 | <0.0001*^a^ |  |
| Post hoc^b^ | CRSwoAR vs Control, P>0.9999; CRSwAR vs. Control, CRSwoAR vs. CRSwAR, P<0.0001 | | | | |
| IgG2a 𝚞g/ml | 49.69±4.74 | 72.32±4.00 | 38.34±4.45 | <0.0001*^a^ |  |
| Post hoc^b^ | CRSwoAR vs Control, P=0.0179*; CRSwoAR vs. control, P=0.4184; CRSwoAR vs. CRSwAR, P<0.0001 | | | | |

CRSwoAR = chronic rhinosinusitis without allergic rhinitis; CRSwAR = chronic rhinosinusitis with allergic rhinitis; IgE =immunoglobulin E; HDM = house dust mite; IgG2a = immunoglobulin G2a; ^a^ Kruskal-Wallis test; ^b^ Dunn’s multiple comparisons test; * P<0.05.

Supplementary table 5. Cytokine levels of experimental chronic rhinosinusitis with or without allergic rhinitis.

| Group | Control (N=6) | CRSwoAR (N=6) | CRSwAR (N=6) | P value |
| --- | --- | --- | --- | --- |
| IL-4 pg/ml  (mean ± SEM) | 1.09±0.26 | 1.53±0.19 | 3.68±0.27 | 0.0002*^a^ |
| Post hoc^b^ | CRSwoAR vs Control, P>0.9999; CRSwAR vs. Control, P =0.0024*; CRSwoAR vs. CRSwAR, P =0.0383* | | | |
| IL-5 pg/ml | 0.78±0.08 | 1.27±0.34 | 1.90±0.41 | 0.0251*^a^ |
| Post hoc^b^ | CRSwoAR vs Control, P=0.8713; CRSwAR vs. Control, P=0.0278*; CRSwoAR vs. CRSwAR, P=0.3670 | | | |
| IL-10 pg/ml | 5.63±0.97 | 4.26±1.00 | 4.07±0.91 | 0.5652^a^ |
| Post hoc^b^ | CRSwoAR vs Control, CRSwoAR vs. CRSwAR, both P>0.9999; CRSwAR vs. Control, P=0.8143 | | | |
| IFN-𝛾 pg/ml | 7.82±2.06 | 5.38±1.67 | 4.36±1.20 | 0.5281^a^ |
| Post hoc^b^ | CRSwoAR vs Control, CRSwoAR vs. CRSwAR, both P>0.9999; CRSwAR vs. Control, P=0.7670 | | | |
| IL-6 pg/ml | 2.42±0.51 | 2.1±0.46 | 1.89±0.40 | 0.7975^a^ |
| Post hoc^b^ | CRSwoAR vs Control, CRSwAR vs. Control, CRSwoAR vs. CRSwAR, all P>0.9999 | | | |
| IL17A pg/ml | 3.63±0.88 | 2.98±0.45 | 2.76±0.32 | 0.8679^a^ |
| Post hoc^b^ | CRSwoAR vs Control, CRSwAR vs. Control, CRSwoAR vs. CRSwAR, all P>0.9999 | | | |

CRSwoAR = chronic rhinosinusitis without allergic rhinitis; CRSwAR = chronic rhinosinusitis with allergic rhinitis; ^a^ Kruskal-Wallis test; ^b^ Dunn’s multiple comparisons test; P<0.05.

Supplementary Table 6. Relative protein expression of nasal-associated lymphoid tissue from experimental chronic rhinosinusitis mice with or without allergic rhinitis.

| Group | Control (N=17) | CRSwoAR (N=16) | CRSwAR (N=24) | P value |  |
| --- | --- | --- | --- | --- | --- |
| MUC5AC  (mean ± SEM) | 0.72±0.10 | 1.03±0.10 | 1.33±0.16 | 0.0178*^a^ |  |
| Post hoc^b^ | CRSwoAR vs Control, P=0.2319; CRSwAR vs. Control, P = 0.0144*; CRSwoAR vs. CRSwAR, P >0.9999 | | | | |
| E-cadherin | 0.55±0.09 | 0.46±0.08 | 0.46±0.09 | 0.5680^a^ |  |
| Post hoc^b^ | CRSwoAR vs Control, CRSwAR vs. CRSwAR, both P>0.9999; CRSwoAR vs. control, P =0.8633 | | | | |
| Claudin-1 | 0.67±0.07 | 0.77±0.07 | 0.85±0.07 | 0.2731^a^ |  |
| Post hoc^b^ | CRSwoAR vs Control, CRSwAR vs. Control, both P>0.9999; CRSwoAR vs. control, P =0.3227 | | | | |

CRSwoAR = chronic rhinosinusitis without allergic rhinitis; CRSwAR = chronic rhinosinusitis with allergic rhinitis; ^a^ Kruskal-Wallis test; ^b^ Dunn’s multiple comparisons test; P<0.05.

Supplementary Table 7. Immunoglobulin levels of experimental chronic rhinosinusitis mice without allergic rhinitis after erythromycin treatment.

| Group | Chronic rhinosinusitis without allergic rhinitis | | | | |  |
| --- | --- | --- | --- | --- | --- | --- |
| Treatment | - | Erythromycin | | | Dexamethasone |  |
| Dose | - | 0.75 mg/kg | 7.5 mg/kg | 75 mg/kg | 1mg/kg | P value |
|  | N=19 | N =11 | N=11 | N=13 | N=8 |  |
| Total IgE  𝚞g/ml | 1.29±0.30 | 0.62±0.24 | 1.11±0.02 | 1.09±0.01 | 0.61±0.42 | 0.1008 |
| Post hoc^b^ | CRSwoAR vs. EM 0.75, CRSwoAR vs. EM 7.5, CRSwoAR vs. EM 75, CRSwoAR vs. Dex, EM 0.75 vs. Dex, EM 7.5 vs EM 75, all p >0.9999; EM 0.75 vs. EM 7.5, P =0.5407; EM 0.75 vs. EM 75, P = 0.7457; EM 7.5 vs. Dex, P = 0.3410; EM 75 vs. Dex, P = 0.4672 | | | | | |
| HDM IgE  O.D. | 0.16±0.01 | 0.18±0.01 | 0.17±0.03 | 0.16±0.02 | 0.17±0.01 | 0.6213 |
| Post hoc^b^ | All P >0.9999 | | | | | |
| IgG2a  𝚞g/ml | 72.32±3.98 | 71.93±6.11 | 26.71±0.48 | 26.64±0.45 | 36.11±6.90 | <0.0001*^a^ |
| Post hoc^b^ | CRSwoAR vs. EM 0.75, EM 0.75 vs. EM75, EM 7.5 vs. Dex, EM75 vs. Dex, all P >0.9999; CRSwoAR vs. EM 7.5, CRSwoAR vs. EM 75, both P <0.001*; CRSwoAR vs. Dex, P =0.0229*, EM0.75 vs. EM 7.5, P =0.0004*; EM0.75 vs. EM75, P =0.0001*; EM0.75 vs. Dex, P =0.0456* | | | | | |

CRSwoAR = chronic rhinosinusitis without allergic rhinitis; IgE =immunoglobulin E; HDM = house dust mite; IgG2a = immunoglobulin G2a; ^a^ Kruskal-Wallis test; ^b^ Dunn’s multiple comparisons test; * P<0.05.

Supplementary Table 8. Immunoglobulin levels of experimental chronic rhinosinusitis mice with alle

rgic rhinitis after erythromycin treatment.

| Group | Chronic rhinosinusitis with allergic rhinitis | | | | | P value |
| --- | --- | --- | --- | --- | --- | --- |
| Treatment | - | Erythromycin | | | Dex |  |
| Dose | - | 0.75 mg/kg | 7.5 mg/kg | 75 mg/kg | 1 mg/kg |  |
|  | N =27 | N =19 | N=11 | N=12 | N=17 |  |
| Total IgE  𝚞g/ml | 39.86±4.00 | 28.2±2.59 | 20.24±5.35 | 24.25±4.84 | 23.27±3.67 | 0.0039*^a^ |
| Post hoc^b^ | CRSwAR vs. EM0.75, EM0.75 vs. EM75, EM0.75 vs. Dex, EM 7.5 vs. EM75, EM 7.5 vs. Dex, EM75 vs. Dex, all P >0.9999; CRSwAR vs. EM7.5, P =0.0141*; CRSwAR vs. EM75, P =0.0940; CRSwoAR vs. Dex, P=0.0433*; EM0.75 vs. EM7.5, P=0.7180 | | | | | |
| HDM IgE  O.D. | 0.42±0.05 | 0.41±0.04 | 0.39±0.05 | 0.38±0.03 | 0.40±0.04 | 0.9777^a^ |
| Post hoc^b^ | All P>0.9999 | | | | | |
| IgG2a  𝚞g/ml | 38.24±4.45 | 54.25±6.26 | 19.05±1.34 | 17.45±1.99 | 43.84±6.22 | <0.0001*^a^ |
| Post hoc^b^ | CRSwAR vs. EM0.75, CRSwAR vs. Dex, EM0.75 vs. Dex, EM 7.5 vs. EM75, all P >0.9999; CRSwAR vs. EM7.5, P =0.0596; CRSwAR vs. EM 75, P =0.0272*; EM 0.75 vs. EM 7.5 P=0.0009*; EM 0.75 vs. EM75, P=0.0003*; EM7.5 vs. Dex, P =0.0167*; EM75 vs. Dex, P = 0.0075* | | | | | |

CRSwoAR = chronic rhinosinusitis without allergic rhinitis; CRSwAR = chronic rhinosinusitis with allergic rhinitis; IgE =immunoglobulin E; HDM = house dust mite; IgG2a = immunoglobulin G2a; ^a^ Kruskal-Wallis test; ^b^ Dunn’s multiple comparisons test; P<0.05.

Supplementary Table 9. Cytokine levels of experimental chronic rhinosinusitis mice without allergic rhinitis after erythromycin treatment.

| Group | Chronic rhinosinusitis without allergic rhinitis | | | | |  |
| --- | --- | --- | --- | --- | --- | --- |
| Treatment | - | Erythromycin | | | Dexamethasone |  |
| Dose | - | 0.75 mg/kg | 7.5 mg/kg | 75 mg/kg | 1mg/kg | P value |
|  | N= 6 | N= 6 | N = 6 | N = 6 | N = 6 |  |
| IL-4 pg/ml | 1.53±0.19 | 1.36±0.18 | 1.76±0.19 | 1.51±0.10 | 1.31±0.33 | 0.6242^a^ |
| Post hoc^b^ | All p >0.9999 | | | | | |
| IL-5 pg/ml | 1.27±0.34 | 0.69±0.12 | 2.19±0.37 | 2.12±0.25 | 0.70±0.12 | 0.0014*^a^ |
| Post hoc^b^ | CRSwoAR vs. EM 0.75, CRSwoAR vs. Dex, EM 0.75 vs. Dex, EM 7.5 vs. EM 75, all P >0.9999; CRSwoAR vs. EM 7.5, P=0.8326; CRSwoAR vs. EM 75, P =0.7739; EM 0.75 vs. EM 7.5, P =0.0265*; EM 0.75 vs. EM75, P =0.0237*; EM 7.5 vs. Dex, P =0.0395*; EM 75 vs. Dex, P =0.0356* | | | | | |
| IL-10 pg/ml | 4.26±1.00 | 3.62±0.87 | 12.93±0.65 | 11.10±0.38 | 2.85±0.89 | 0.0002* |
| Post hoc^b^ | CRSwoAR vs. EM 0.75, EM 0.75 vs. Dex, EM 7.5 vs. EM 75, all P >0.9999; CRSwoAR vs. EM 7.5, P=0.0387*; CRSwoAR vs. EM 75, P =0.4494; EM 0.75 vs. EM 7.5, P =0.0046*; EM 0.75 vs. EM75, P =0.0887; EM 7.5 vs. Dex, P =0.0021*; EM 75 vs. Dex, P =0.0417* | | | | | |
| IFN-𝛾 pg/ml | 5.38±1.67 | 4.22±1.39 | 16.92±0.48 | 17.65±1.75 | 3.26±1.68 | 0.0002*^a^ |
| Post hoc^b^ | CRSwoAR vs. EM 0.75, CRSwoAR vs. Dex, EM 0.75 vs. Dex, EM 7.5 vs. EM 75, all P >0.9999; CRSwoAR vs. EM 7.5, P=0.1264; CRSwoAR vs. EM 75, P =0.1676; EM 0.75 vs. EM 7.5, P =0.0253*; EM 0.75 vs. EM75, P =0.0352*; EM 7.5 vs. Dex, P =0.0061*; EM 75 vs. Dex, P =0.0087* | | | | | |
| IL-6 pg/ml | 2.10±0.46 | 1.70±0.40 | 3.48±0.21 | 3.19±0.22 | 1.72±0.37 | 0.0092*^a^ |
| Post hoc^b^ | CRSwoAR vs. EM 0.75, CRSwoAR vs. Dex, EM 0.75 vs. Dex, EM 7.5 vs. EM 75, all P >0.9999; CRSwoAR vs. EM 7.5, P=0.3267; CRSwoAR vs. EM 75, P =0.8684; EM 0.75 vs. EM 7.5, P =0.0374*; EM 0.75 vs. EM75, P =0.1331; EM 7.5 vs. Dex, P =0.1339; EM 75 vs. Dex, P =0.3854 | | | | | |
| IL-17 pg/ml | 2.98±0.45 | 2.41±0.36 | 4.89±0.30 | 4.61±0.30 | 3.49±1.14 | 0.0080*^a^ |
| Post hoc^b^ | CRSwoAR vs. EM 0.75, CRSwoAR vs. Dex, EM 0.75 vs. Dex, EM 7.5 vs. EM 75, all P >0.9999; CRSwoAR vs. EM 7.5, P=0.1389; CRSwoAR vs. EM 75, P =0.3394; EM 0.75 vs. EM 7.5, P =0.0372*; EM 0.75 vs. EM75, P =0.1041; EM 7.5 vs. Dex, P =0.3491; EM 75 vs. Dex, P =0.7411 | | | | | |

CRSwoAR = chronic rhinosinusitis without allergic rhinitis; CRSwAR = chronic rhinosinusitis with allergic rhinitis; ^a^ Kruskal-Wallis test; ^b^ Dunn’s multiple comparisons test; P<0.05.

Supplementary Table 10. Cytokine levels of experimental chronic rhinosinusitis mice with allergic rhinitis after erythromycin treatment.

| Group | Chronic rhinosinusitis with allergic rhinitis | | | | | P value |
| --- | --- | --- | --- | --- | --- | --- |
| Treatment | - | Erythromycin | | | Dex |  |
| Dose | - | 0.75 mg/kg | 7.5 mg/kg | 75 mg/kg | 1 mg/kg |  |
|  | N=6 | N=6 | N=6 | N=6 | N=6 |  |
| IL-4 pg/ml | 3.68±0.27 | 2.86±0.31 | 5.00±0.89 | 5.19±1.08 | 2.86±0.39 | 0.1006^a^ |
| Post hoc^b^ | CRSwAR vs. EM 0.75, CRSwAR vs. EM7.5, CRSwAR vs. EM75, CRSwAR vs. Dex, EM 0.75 vs. Dex, EM 7.5 vs. 75, all P >0.9999; EM 0.75 vs. 7.5, P =0.3469; EM 0.75 vs. 75, P =0.6782; EM 7.5 vs. Dex, P =0.4195; EM 75 vs. Dex, P =0.8232 | | | | | |
| IL-5 pg/ml | 1.90±0.41 | 1.00±0.25 | 1.71±0.24 | 1.89±0.48 | 0.88±0.16 | 0.0726^a^ |
| Post hoc^b^ | CRSwAR vs. EM0.7.5, CRSwAR vs. EM75, EM0.75 vs. EM75, EM0.75 vs. Dex, EM 7.5 vs. Dex, all P >0.9999; CRSwAR vs. EM0.75, P =0.6968; CRSwAR vs. Dex, P =0.2758; EM 0.75 vs. EM 7.5 P=0.9167; EM 7.5 vs. Dex, P=0.3834; EM75 vs. Dex, P =0.6577 | | | | | |
| IL-10 pg/ml | 4.07±0.91 | 3.79±1.12 | 11.28±0.38 | 11.05±0.53 | 3.46±1.03 | 0.0002*^a^ |
| Post hoc^b^ | CRSwAR vs. EM0.75, CRSwAR vs. Dex, EM0.75 vs. Dex, EM 7.5 vs. EM75, all P >0.9999; CRSwAR vs. EM7.5, P =0.0994; CRSwAR vs. EM 75, P =0.1426; EM 0.75 vs. EM 7.5 P=0.0237*; EM 0.75 vs. EM75, P=0.0359*; EM7.5 vs. Dex, P =0.0043*; EM75 vs. Dex, P = 0.0070* | | | | | |
| IFN-𝛾 pg/ml | 4.36±1.20 | 4.59±1.38 | 16.24±1.05 | 15.19±1.15 | 3.67±0.97 | 0.0002*^a^ |
| Post hoc^b^ | CRSwAR vs. EM0.75, CRSwAR vs. Dex, EM 0.75 vs. Dex, EM7.5 vs. EM75, all P >0.99; CRSwAR vs. EM7.5, P =0.0281*; CRSwAR vs. EM 75, P =0.1003; EM 0.75 vs. EM 7.5 P=0.0444*; EM 0.75 vs. EM75, P=0.1502; EM7.5 vs. Dex, P =0.0020*; EM75 vs. Dex, P = 0.0100* | | | | | |
| IL-6 pg/ml | 1.89±0.40 | 1.54±0.30 | 4.09±0.45 | 3.15±0.27 | 1.41±0.22 | 0.0008*^a^ |
| Post hoc^b^ | CRSwAR vs. EM0.75, CRSwAR vs. Dex, EM0.75 vs. Dex, EM7.5 vs. EM75, all P >0.9999; CRSwAR vs. EM7.5, P =0.0471*; CRSwAR vs. EM 75, P =0.6552; EM 0.75 vs. EM 7.5 P=0.0196*; EM 0.75 vs. EM75, P=0.3472; EM7.5 vs. Dex, P =0.0028*; EM75 vs. Dex, P = 0.0899 | | | | | |
| IL-17A pg/ml | 2.76±0.32 | 2.87±0.42 | 4.51±0.34 | 4.02±0.21 | 2.40±0.22 | 0.0018*^a^ |
| Post hoc^b^ | CRSwAR vs. EM0.75, CRSwAR vs. Dex, EM 0.75 vs. Dex, EM7.5 vs. EM75, all P >0.9999; CRSwAR vs. EM7.5, P =0.0961; CRSwAR vs. EM 75, P =0.3456; EM 0.75 vs. EM 7.5 P=0.1319; EM 0.75 vs. EM75, P=0.4530; EM7.5 vs. Dex, P =0.0069*; EM75 vs. Dex, P = 0.0377* | | | | | |

Control=non-chronic rhinosinusitis, non-allergic rhinitis mice; CRSwoAR =chronic rhinosinusitis without allergic rhinitis mice; CRSwAR = chronic rhinosinusitis with allergic rhinitis mice; ^a^ Kruskal-Wallis test; ^b^ Dunn’s multiple comparisons test; P<0.05.

Supplementary Table 11. Relative protein expression of nasal-associated lymphoid tissue from experimental chronic rhinosinusitis mice without allergic rhinitis.

| Group | Chronic rhinosinusitis without allergic rhinitis | | | | |  |
| --- | --- | --- | --- | --- | --- | --- |
| Treatment | - | Erythromycin | | | Dexamethasone |  |
| Dose | - | 0.75 mg/kg | 7.5 mg/kg | 75 mg/kg | 1mg/kg | P value |
|  | N= 16 | N= 12 | N = 12 | N = 13 | N = 9 |  |
| MUC5AC  (mean ± SEM) | 1.03±0.10 | 1.22±0.20 | 0.34±0.04 | 0.43±0.07 | 0.88±0.17 | <0.0001^a^ |
| Post hoc^b^ | CRSwoAR. Vs EM 0.75, CRSwoAR vs. Dex, EM 0.75 vs. Dex, EM 7.5 vs. EM 75, all P>0.9999; CRSwoAR, vs. EM 7.5, P =0.0003*; CRSwoAR vs. EM 75, P = 0.0032*; EM 0.75 vs. EM 7.5, P =0.0004*; EM 0.75 vs. EM 75, P = 0.0042*; EM 7.5 vs. Dex, P = 0.0529; EM 75 vs. Dex, P = 0.2470 | | | | | |
| E-cadherin | 0.46±0.08 | 0.18±0.05 | 0.71±0.16 | 0.86±0.13 | 0.27±0.12 | 0.0012*^a^ |
| Post hoc^b^ | CRSwoAR vs. EM 7.5, CRSwoAR vs. Dex, EM 0.75 vs. Dex, EM 7.5 vs. EM 75, all P >0.9999; CRSwoAR vs. EM 0.75, P =0.3686; CRSwoAR vs. EM 75, P =0.5301; EM 0.75 vs. EM 7.5, P =0.0957; EM 0.75 vs. EM75, P =0.0015*; EM 7.5 vs. Dex, P =0.6021; EM 75 vs. Dex, P =0.0293* | | | | | |
| Claudin-1 | 0.77±0.07 | 0.79±0.12 | 0.80±0.10 | 0.80±0.06 | 0.72±0.09 | 0.9460^a^ |
| Post hoc^b^ | All P>0.9999 | | | | | |

CRSwoAR = chronic rhinosinusitis without allergic rhinitis; ^a^ Kruskal-Wallis test; ^b^ Dunn’s multiple comparisons test; P<0.05.

Supplementary Table 12. Relative protein expression of nasal-associated lymphoid tissue from experimental chronic rhinosinusitis mice with allergic rhinitis.

| Group | Chronic rhinosinusitis with allergic rhinitis | | | | |  |
| --- | --- | --- | --- | --- | --- | --- |
| Treatment | - | Erythromycin | | | Dexamethasone |  |
| Dose | - | 0.75 mg/kg | 7.5 mg/kg | 75 mg/kg | 1mg/kg | P value |
|  | N= 24 | N= 15 | N = 12 | N = 13 | N = 17 |  |
| MUC5AC  (mean ± SEM) | 1.33±0.16 | 0.90±0.20 | 0.41±0.06 | 0.58±0.07 | 0.95±0.12 | <0.0001*^a^ |
| Post hoc^b^ | CRSwAR. Vs EM 0.75, CRSwAR vs. Dex, EM 0.75 vs. Dex, EM 7.5 vs. EM 75, all P>0.9999; CRSwAR, vs. EM 7.5, P <0.0001*; CRSwAR vs. EM 75, P = 0.0134*; EM 0.75 vs. EM 7.5, P =0.0254*; EM 0.75 vs. EM 75, P = 0.7312; EM 7.5 vs. Dex, P = 0.0190*; EM 75 vs. Dex, P = 0.6465 | | | | | |
| E-cadherin | 0.46±0.08 | 0.57±0.11 | 0.86±0.21 | 0.84±0.07 | 0.35±0.06 | 0.0060*^a^ |
| Post hoc^b^ | CRSwAR vs. EM 0.75, CRSwAR vs. Dex, EM 0.75 vs. EM 7.5, EM 0.75 vs. Dex, EM 7.5 vs. EM 75, all P >0.9999; CRSwAR vs. EM 7.5, P =0.7698; CRSwAR vs. EM 75, P =0.0178*; EM 0.75 vs. EM 7.5, P =0.5278; EM 7.5 vs. Dex, P =0.4474; EM 75 vs. Dex, P =0.0105* | | | | | |
| Claudin-1 | 0.85±0.07 | 0.79±0.06 | 0.72±0.06 | 0.89±0.08 | 0.85±0.08 | 0.7627^a^ |
| Post hoc^b^ | All P>0.9999 | | | | | |

CRSwAR = chronic rhinosinusitis with allergic rhinitis; ^a^ Kruskal-Wallis test; ^b^ Dunn’s multiple comparisons test; P<0.05.
